# Hydrogel-mediated extracellular vesicles for enhanced wound healing: the latest progress, and their prospects for 3D bioprinting

**DOI:** 10.1186/s12951-024-02315-9

**Published:** 2024-02-10

**Authors:** Yi Zheng, Chuqiao Pan, Peng Xu, Kai Liu

**Affiliations:** Department of Plastic and Reconstructive Surgery, Shanghai Ninth People’s Hospital, Shanghai Jiao Tong University School of Medicine, 639 Zhi Zao Ju Road, Shanghai, 200011 China

**Keywords:** Extracellular vesicle, Hydrogels, Would healing, 3D bioprinting, Regenerative medicine

## Abstract

Extracellular vesicles have shown promising tissue recovery-promoting effects, making them increasingly sought-after for their therapeutic potential in wound treatment. However, traditional extracellular vesicle applications suffer from limitations such as rapid degradation and short maintenance during wound administration. To address these challenges, a growing body of research highlights the role of hydrogels as effective carriers for sustained extracellular vesicle release, thereby facilitating wound healing. The combination of extracellular vesicles with hydrogels and the development of 3D bioprinting create composite hydrogel systems boasting excellent mechanical properties and biological activity, presenting a novel approach to wound healing and skin dressing. This comprehensive review explores the remarkable mechanical properties of hydrogels, specifically suited for loading extracellular vesicles. We delve into the diverse sources of extracellular vesicles and hydrogels, analyzing their integration within composite hydrogel formulations for wound treatment. Different composite methods as well as 3D bioprinting, adapted to varying conditions and construction strategies, are examined for their roles in promoting wound healing. The results highlight the potential of extracellular vesicle-laden hydrogels as advanced therapeutic tools in the field of wound treatment, offering both mechanical support and bioactive functions. By providing an in-depth examination of the various roles that these composite hydrogels can play in wound healing, this review sheds light on the promising directions for further research and development. Finally, we address the challenges associated with the application of composite hydrogels, along with emerging trends of 3D bioprinting in this domain. The discussion covers issues such as scalability, regulatory considerations, and the translation of this technology into practical clinical settings. In conclusion, this review underlines the significant contributions of hydrogel-mediated extracellular vesicle therapy to the field of 3D bioprinting and wound healing and tissue regeneration. It serves as a valuable resource for researchers and practitioners alike, fostering a deeper understanding of the potential benefits, applications, and challenges involved in utilizing composite hydrogels for wound treatment.

**Graphical abstract:**

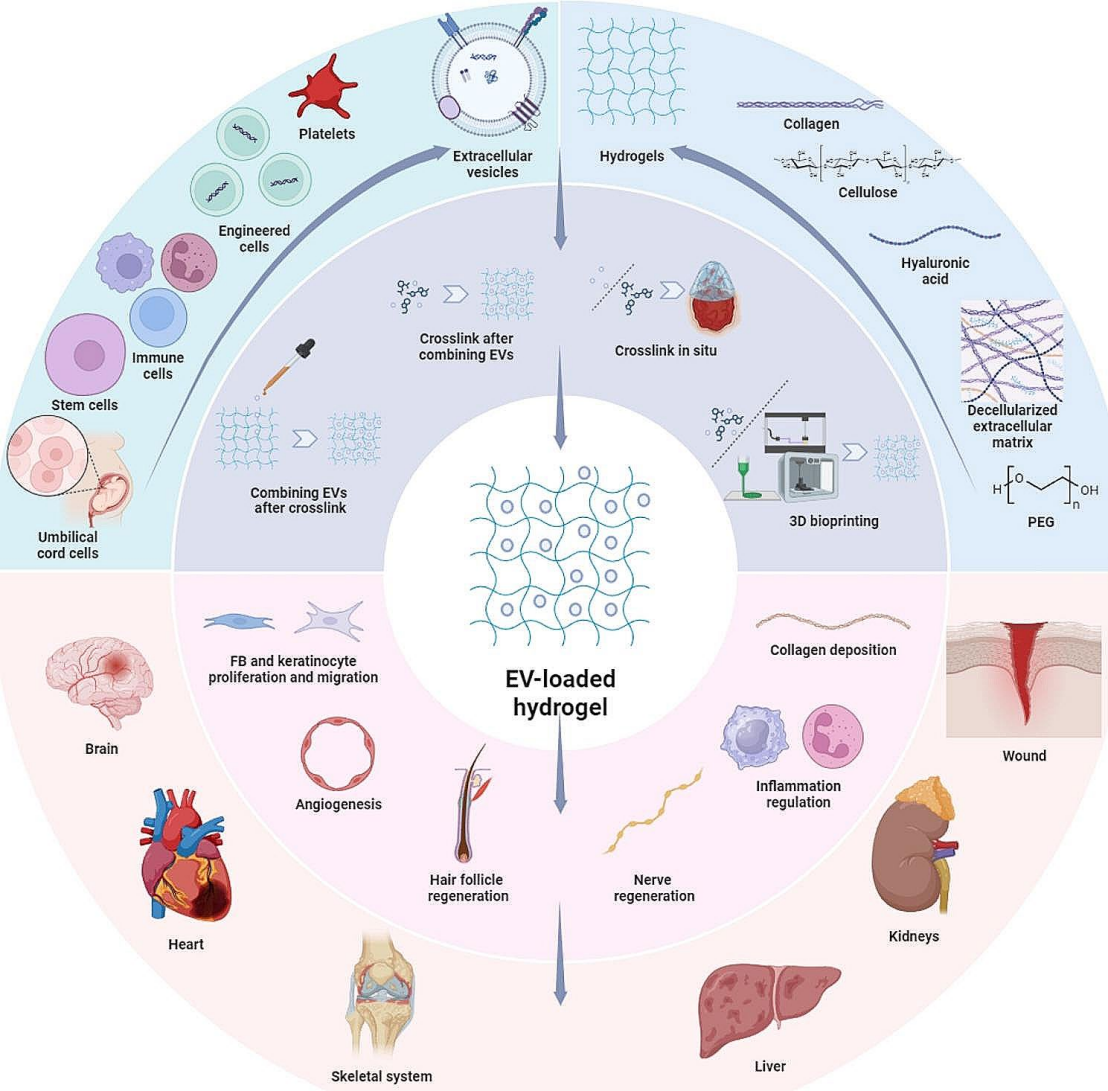

## Introduction

Cutaneous wound healing is an intricately regulated process comprised of four continuous and overlapping phases: hemostasis, inflammation, proliferation, and remodeling. After skin injury, injured blood vessels undergo reactive constriction, and a red thrombus is formed through the coordinated activation of platelets and the coagulation cascade, achieving initial hemostasis [1]. In the subsequent inflammatory phase, neutrophils, and monocytes which differentiate into M1 (pro-inflammatory) macrophages, engage in phagocytosis of pathogens and tissue debris. Concurrently, they secrete various chemotactic and cytokine factors, mediating localized immune responses [2]. The hallmark of the proliferation phase is characterized by accelerated angiogenesis and collagen deposition. M1 macrophages shift to a M2 (pro-healing) phenotype, and facilitate fibroblast, endothelial cell, and keratinocyte proliferation, differentiation, and migration, culminating in the formation of a complete epithelial layer and subepidermal tissue [3]. The final remodeling phase is marked by collagen restructuring in the wound, accompanied by regression of excess vessels and apoptosis of surplus cells, resulting in scar formation [4].

However, various etiological factors such as diabetes and vascular diseases may lead to delayed or stalled healing processes, resulting in chronic wounds, which poses a significant clinical challenge [5]. Despite substantial research, the development of innovative wound healing strategies remains a top priority [6, 7]. Recent advances in research have led to the development of various innovative approaches, with stem cell therapy, especially utilizing mesenchymal stem cells (MSCs), emerging as a prominent choice. MSCs, known for their self-renewal and differentiation capabilities, exhibit therapeutic potential in wound healing [8, 9]. However, extracellular vesicles (EVs), crucial components of MSC paracrine products, have been found to be the key mediators of MSC therapeutic effects [10, 11].

EVs include exosomes, microvesicles (MVs), and apoptotic bodies. They are commonly secreted by various cell types within the body, and their presence is nearly ubiquitous in all bodily fluids [12]. EVs constitute cell-derived nanoparticles surrounded by lipid bilayer membranes that protect their contents, including proteins, nucleic acids, and lipids, from environmental degradation [13]. Initially perceived as cellular waste disposal units, EVs were found to transport bioactive cargo and facilitate cell-to-cell communication [14–16]. Compared to stem cells, EVs offer advantages, including lower tumorigenic risk, and are anticipated to be useful in wound treatment [17]. Studies have highlighted their role in promoting wound healing through mechanisms such as fibroblast and keratinocyte activation, collagen deposition and inflammation regulation [18, 19]. Besides, EVs also play pivotal roles in diverse physiological and pathological processes, including immune modulation, angiogenesis, and tissue repair, offering widespread applications in the treatment of various organ diseases, such as those affecting the brain, heart, skeletal system, liver, kidneys, and skin [20].

However, challenges in EV clinical use include reduced bioavailability and susceptibility to environmental factors. EVs through systemic intravenous injection are easily cleared by the liver and spleen [21] with only approximately 1% EVs remained at 24 h [22]. Local administration faces limitations due to quick elimination after being absorbed into the tissue and blood, necessitating multiple doses and potentially disrupting healing processes. Besides, pH, reactive oxygen species (ROS), and ionic components can also impact EV stability [23].

**Fig. 1:**
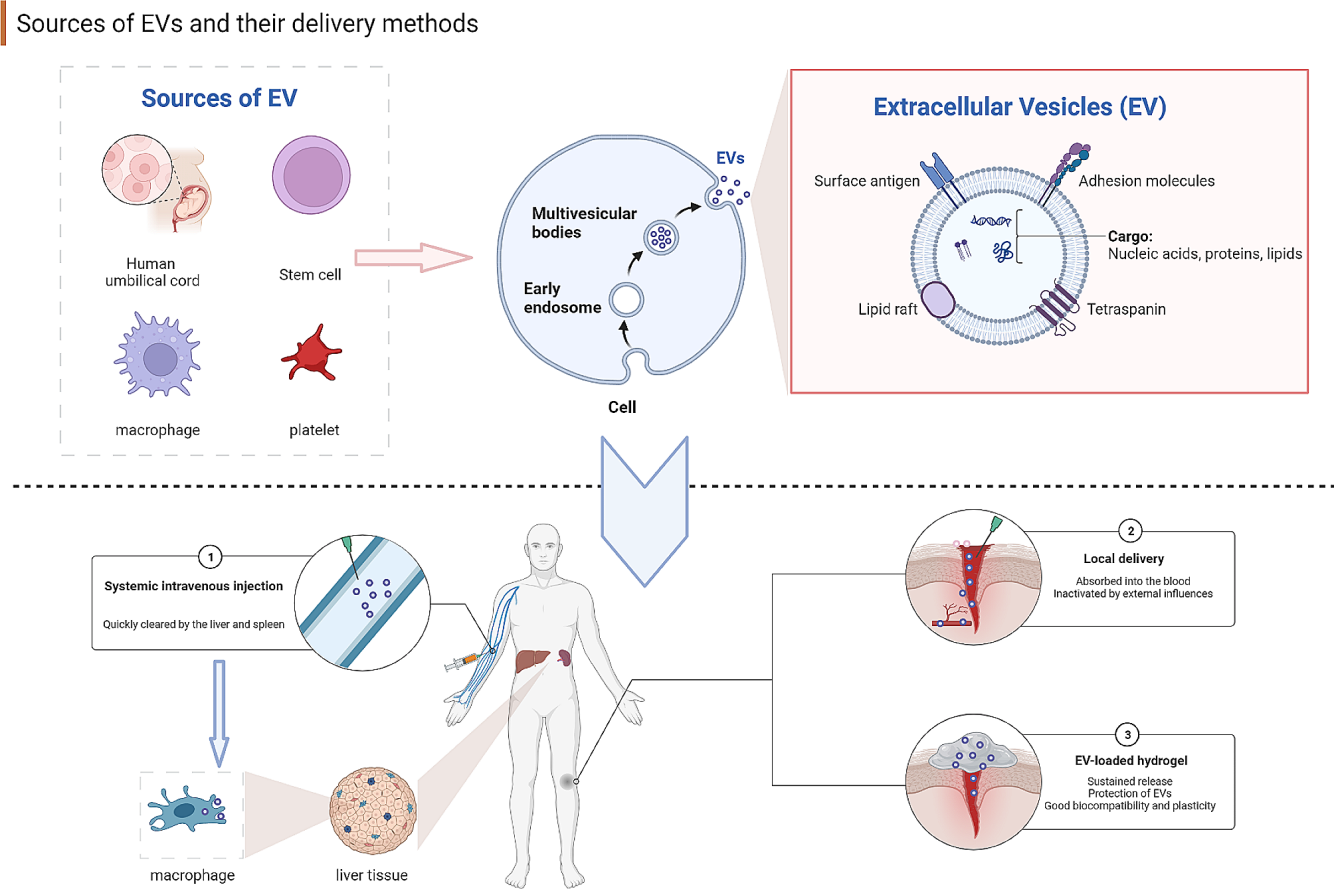
The sources of EVs and their delivery methods. EVs exhibit diverse origins, including umbilical cord-derived cells, stem cells, immune cells, and platelets. Their administration in wound healing can be categorized into systemic and local delivery. Systemic administration faces challenges like quick clearance by liver and spleen macrophages, while local delivery risks absorption into the bloodstream or rapid EV elimination due to external factors. EV-loaded hydrogels can offer protection and sustained release of EVs, and exhibit biocompatibility and malleability, making them a promising strategy for wound healing

To overcome these challenges, 3D materials, particularly hydrogels, have gained attention in wound healing. Hydrogels, 3D polymer networks with high water content, mimic the extracellular matrix (ECM) and have good biocompatibility and plasticity [24, 25]. Their mechanical properties can be tailored for specific applications [26]. Hydrogels exhibit excellent potential as ideal wound dressings, maintaining a pro-healing microenvironment, and as drug delivery systems, protecting and controlling EV release (Fig. 1). As technology advances, 3D bioprinting is highly anticipated in the treatment of wounds. 3D bioprinting is an advanced additive manufacturing technique [27], and can form three-dimensional scaffolds similar to natural human tissues [28]. Combining EVs with hydrogels through 3D bioprinting presents opportunities in regenerative medicine.

This review outlines recent advancements in EV-loaded hydrogels for wound healing, and the prospects for 3D bioprinting. It covers the advantages of the composite hydrogels, EV and hydrogel characteristics, synthesis methods, and the impact of the composite hydrogels on wound healing. The discussion includes existing challenges, future research directions, and implications in the field.

## Advantages of extracellular vesicles-loaded hydrogels

### Powerful EV reserve capacity

Hydrogels are composed of hydrophilic polymer networks that exhibit high swelling properties and can absorb water several thousand times their weight [29]. This ability makes hydrogels similar to the physical properties of normal tissues and provides hydrogels with excellent biocompatibility and the ability to easily encapsulate contents like EVs [30]. In addition, the polymer networks in hydrogels form many micropores [29], providing a large surface area that contributes to the load of EVs [31]. Hydrogels can be designed to further increase absorption efficiency. For example, Ma et al. added fusion peptides, which consisted of collagen-binding domains, to composite hydrogels. They genetically engineered peptides that bind collagen I and III, and CP05, a polypeptide fragment that binds CD63, bound EVs tightly to the hydrogel materials. Compared with ordinary composite hydrogels loaded with EVs by physical adsorption, the engineered hydrogels with fusion peptides loaded more EVs, with more particles released over a longer time [32].

### Protection of EVs

Owing to their swelling properties, hydrogels maintain a humid environment in the wound area by absorbing significant amounts of water, protecting EVs from external environmental damage. Additionally, the crosslinking networks of hydrogels can prevent the penetration of other substances such as enzymes or ions to protect EVs from premature degradation and destruction, which is conducive to the preservation of EV stability and biological functions for a long time [33]. Mardpour et al. designed a composite hydrogel based on clickable polyethylene glycol (PEG) macromeres and found effective EVs released after four weeks [34]. Wu et al. found that EVs, after 28-day storage in gelatin methacryloyl (GelMA) hydrogel at 4 °C, had similar particle numbers, sizes, structures, and protein levels as fresh EVs and still maintained the ability to promote cell proliferation and angiogenesis. This may be due to the slower movement of the EVs and a reduction in their aggregation in the hydrogels [35].

### Controlled release of EVs

Compared to the direct application of EVs, one of the major advantages of EV-loaded hydrogels is their sustained release ability, which avoids multiple drug administration. This effect was based on the porous structure of the hydrogels. EVs can be released rapidly when the pore size of the hydrogel crosslinking networks is larger than the diameter of EVs, whereas EVs remain in situ when the pore size is smaller than that of EVs. With the expansion and degradation of hydrogels in the tissue, the pore size gradually increases, releasing EVs [29]. Therefore, sustained release can be achieved by changing the size of the hydrogel pores or by adjusting the swelling properties and degradation rates of different hydrogels.

Hydrogels can swell, degrade spontaneously, and release EVs into wounds. Hydrogels can be tailored with diverse mechanical properties and degradation rates by adjusting the synthesis of their precursor materials, catering to various release requirements. For instance, Sun et al. developed cryo-self-assembled silk fibroin (SF) sponge hydrogels designed specifically for loading EVs. The degradation rate of the hydrogel could be changed by varying the fibroin concentration. In one study, higher fibroin concentration exhibited a slower degradation rate and a longer EV release time [36]. In addition, hydrogels can be designed based on the wound features. For example, Wang et al. established a controlled-release hydrogel based on the reversible Schiff base bond formed by oxidative hyaluronic acid (OHA) and Poly-ε-L-lysine (EPL). The Schiff base bond can break under weakly acidic conditions so that EVs can be released in the acidic area of wounds to achieve a specific treatment purpose [37]. Moreover, hydrogels can be artificially designed to achieve a controlled release of EVs for specific purposes. Sun et al. devised a composite hydrogel incorporating chitosan nanofibrous microspheres and EVs derived from dental pulp stem cells (DPSCs), enabling the programmed release of vascular endothelial growth factor (VEGF) and DPSCs-EVs. This design mimics the physiological environment and tissue repair processes, offering therapeutic potential [38].

### Excellent wound dressing materials

Hydrogels employed for wound dressing necessitate excellent biocompatibility, and their degradation byproducts should be non-toxic. Currently, numerous natural or synthetic hydrogel biomaterials have found widespread application in various wound healing scenarios. This will be further elaborated in subsequent sections. Besides, ECM provides structural support and biological signaling stimuli for cell growth and tissue development. In damaged tissues, ECM is often lost, leading to a lack of extracellular matrix components essential for cell communication and tissue repair [39]. Hydrogels have physical and biological properties similar to those of the ECM, can serve as substitutes by providing extracellular matrix components, thereby facilitating wound healing. Some hydrogels used alone can promote wound healing [40].

### Adjustable mechanical properties

Hydrogels can change between hydrogel and solid states under disparate external conditions and have a wide range of mechanical properties to accommodate different use scenarios. The stiffness of hydrogels can be adjusted from 0.5 kPa to 5 MPa to fix wound tissues with different hardness [30]. Hydrogels can be designed with shear-thinning properties and can easily adapt to the shape of wounds via in situ gelation after injection [41]. Some hydrogels are made into microneedles to penetrate the skin and deliver drugs [42]. Certain hydrogels possess self-healing capabilities to prevent rupture under external pressure, mitigating the risk of performance loss and potential infection caused by bacterial invasion from the external environment [43].

### Easily engineered for more purposes

For example, Xiang et al. designed a multifunctional hydrogel with protocatechualdehyde component to confer antibacterial ability [44]. Xiong et al. designed a multifunctional hydrogel consisting of HA modified with a quaternary ammonium group, which endowed the hydrogel with antibacterial properties and the ability to promote hemostasis [45]. The OxOBand hydrogel designed by Shiekh et al. contained ascorbic acid in the polyurethane backbone and possessed good antioxidant capacity [46]. Thus, hydrogels can be easily engineered to promote wound healing.

## Components of extracellular vesicles-loaded hydrogels

Advancements in understanding the diverse functions of EVs derived from various cells, along with ongoing research progress on different hydrogels, have led to the combination of multiple EV types with various hydrogel formulations.

### Sources and features of different EVs

EVs from different sources have different secretomes, which affect their biological functions [47]. Table 1 summaries the sources of EVs used in EV-loaded composite hydrogels for wound healing.

**Table 1:** Summary sources of EVs used in EV-loaded composite hydrogels for wound healing

Sources of EVs	Advantages	References
Human umbilical cord (including HUVECs, hUCMSCs, hUCB-MNCs)	Easy to obtain, causes less immune rejection than other cells	[48–52]
BMSCs	Commonly used, low infection rate, low immune rejection reactions, and stable cell culture and biological effects	[36, 53]
ADSCs	Easy to obtain, longer lifespan and higher proliferative capacity	[33]
GMSCs	Easy to obtain, strong proliferation ability and significant biological effects for tissue regeneration and immune regulation	[54]
EPCs	Can accelerate the proliferation, migration, and tube formation of HUVECs as well as the treatment of diabetic wound healing	[55]
Synovium-derived stem cells	Tissue-specific properties, efficient repair of tissues and promote wound healing	[56, 57]
M2 macrophages	Polarization of M1 macrophages to the M2 phenotype, promote wounds to the proliferative phase	[58]
PRP	Cheap, easy to obtain, encapsulate many growth factors from platelets	[59]
Royal jelly	Have antibacterial and regenerative properties, can stimulate MSC migration	[60]
Engineered EVs	Endowed with distinct characteristics through management	[61, 62]

#### Umbilical cord cells

Cells derived from human umbilical cord are commonly used for this purpose. They are easier to obtain because the umbilical cord is considered waste after birth [48] and they causes less immune rejection than other cells [49]. Many cell types are derived from the umbilical cord, including human umbilical vein endothelial cells (HUVECs), human umbilical cord mesenchymal cells (hUCMSCs), and human umbilical cord blood mononuclear cells (hUCB-MNCs). HUVEC-EVs promote the proliferation and migration of keratinocytes and FBs, which are important cells for wound healing [50]. hUCMSC-derived EVs promote angiogenesis and accelerate wound healing [51]. HUCB-MNC-EVs combined with hydrogels promoted wound healing in diabetic mouse models [52].

#### Stem cells

Bone marrow stem cells (BMSCs) are among the most commonly used cell types. They originate from the bone marrow and thus have the advantages of a low infection rate, low immune rejection reactions, and stable cell culture and biological effects [53]. Therefore, they can be used as stable sources of EVs. The application of BMSC-EVs with a sustained-release platform combined with a SF scaffold promotes angiogenesis in nude mouse wounds [36].

Adipose derived stem cells (ADSCs) can be easily obtained at a high yield. Moreover, ADSCs have a longer lifespan and higher proliferative capacity than BMSCs [33] and are suitable for secreting EVs. Applications of ADSC-EVs combined with hydrogels have also been investigated. For example, Zhou et al. compounded ADSC-EVs with a Pluronic F127 hydrogel to promote wound healing via collagen deposition, angiogenesis and inflammation control [33].

Various other cells are available for EV production, including gingival stem cells (GMSCs) and epidermal stem cells (EPCs). GMSCs are easily obtained with strong proliferation ability and significant biological effects for tissue regeneration and immune regulation, and are reported to promote wound healing [54]. EPCs have also been reported to accelerate the proliferation, migration, and tube formation of HUVECs as well as the treatment of diabetic wound healing [55]. Synovium-derived stem cells have tissue-specific properties that lead to efficient repair of tissues [56], and are confirmed to aid wound healing [57].

#### Immune cells

EVs derived from immune cells have garnered significant attention due to their distinct characteristics, particularly their ability to regulate inflammation. Kwak et al. combined EVs from M2 macrophages with degradable PEG hydrogels in wounds. These EVs induced the polarization of M1 macrophages to the M2 phenotype and promoted the transformation of wounds from the inflammatory to the proliferative phase, which has clear advantages in early wound healing [58].

#### Noncellular biological components

EVs derived from noncellular components are also utilized. For example, EVs derived from the widely used platelet-rich plasma (PRP) in clinical practice are highly anticipated as PRP is cheap and easy to obtain, and PRP-EVs encapsulate many growth factors from platelets. Xu et al. used PRP-EVs in combination with a chitosan/silk hydrogel and found that it effectively promoted the healing of diabetes wounds [59].

#### Non-human biological components

EVs from non-human sources are also an option. Ramírez et al. combined EVs from royal jelly with type I collagen hydrogels for wound healing. It has been reported that royal jelly has antibacterial and regenerative properties and can stimulate MSC migration. The composite hydrogel promotes the proliferation and migration of FBs [60].

#### Manually engineered biological components

Lastly, EVs derived from cells after special treatments or manually engineered EVs can be utilized. They are endowed with distinct characteristics through management. For example, EVs from ADSCs subjected to hypoxia exhibit pro-restorative properties. Hu et al. designed a composite GelMA hydrogel loaded with EVs for wound treatment, which promoted the migration and tube formation of HUVECs and accelerated diabetic wound healing through collagen deposition and angiogenesis [61]. Synovium-derived stem cells can strongly promote FB proliferation but cannot promote revascularization. Tao et al. transfected EVs derived from synovial stem cells with miR-126, which plays a role in the angiogenic ability of endothelial progenitor cells. A chitosan hydrogel loaded with modified EVs promoted wound healing and angiogenesis [62].

### Sources and features of different hydrogels

Hydrogels can be divided into natural and synthetic hydrogels. Natural hydrogels were the first to be studied and have since been widely used. They possess numerous advantages, including excellent biological activity and biocompatibility, as they are commonly found in biological tissues, and their degradation products are predominantly non-toxic [63]. Commonly used natural-origin hydrogels include single ingredients such as alginate, collagen, chitosan (CS), cellulose, silk fibroin, gelatin, and hyaluronic acid (HA), or complex ingredients such as the small intestinal submucosa and decellularized extracellular matrix (dECM) [33]. Synthetic hydrogels are gaining popularity owing to the availability of cheaper and more reliable raw materials with production stability. In addition, they can be customized with a wide range of physical characteristics to meet the different demands of wound dressing [64]. Synthetic polymers can be used alone or incorporated into natural hydrogels if wound dressings require better mechanical properties or more effective manufacturing capabilities [65]. Commonly used synthetic hydrogels include polyvinyl alcohol (PVA), polyurethane (PU), PEG, polycaprolactone (PCL), and Pluronic F127. Table 2 summaries the materials used in EV-loaded composite hydrogels for wound healing.

**Table 2:** Summary materials used in EV-loaded composite hydrogels for wound healing

Materials	Advantages	References
Alginate	The most prevalent natural biopolymers, good biocompatibility, non-toxicity, low extraction cost, ease of gelation, high absorbency	[66]
Collagen	An abundant natural protein in the body, high water absorbency, enabling printing through precise control of pH and temperature, a natural receptor for cellular adhesion	[60]
Chitosan	High biocompatibility and biodegradability, antimicrobial properties, sustaining cell growth and proliferation, activating macrophages and inducing the release of cytokines, suitable for 3D bioprinting applications	[43, 62]
Cellulose	Diverse and tunable mechanical, structural, chemical, and physical properties, easily obtainable and cost-effective, high biocompatibility, biodegradability, and moisturizing attributes, facilitating the migration and proliferation of dermal fibroblasts while inhibiting bacterial proliferation	[43]
Silk fibroin	Excellent biocompatibility and biodegradability, promoting wound cell growth, differentiation, and extracellular matrix regeneration, mimicking the mechanical behavior of normal tissues	[36, 67]
Gelatin	Excellent biocompatibility, non-immunogenicity, good cell affinity, and degrades completely in vivo, promoting cell adhesion, thermosensitivity	[55]
Hyaluronic acid	The most common component in the ECM, promoting cell adhesion, migration, and proliferation, water-soluble property, suitable for bioprinting	[68]
Decellularized extracellular matrix	Preserving the structural and functional features of the ECM, facilitating cellular processes like adhesion and proliferation	[69]
PVA	Ionic responsiveness and easily tunable mechanical strengths	[70]
PU	Biodegradability and highly porous cryogels providing a matrix for cell migration	[46]
PEG	Great biocompatibility and biodegradability	[71]
PCL	Sufficient mechanical strength, easy for 3D bioprinting	[72]
PF127	Biocompatible, mild inflammatory properties, absorbing the secretions from the wound surface, maintaining a moist environment, heat-sensitive and injectable properties	[37, 73]

In this review, our emphasis centers on the most prevalent and representative natural biomaterials employed for 3D bioprinting bioinks, particularly polysaccharides materials, and their multifaceted functionalities in wound healing and tissue regeneration.

#### Alginate

Recognized as one of the most prevalent natural biopolymers [74], alginate, a carbohydrate extracted from brown algae, stands out as an exceptional choice for bioink design and tissue engineering, offering distinctive advantages. Resembling the extracellular matrix in structure, alginate facilitates efficient cellular oxygen and nutrient exchange, boasts biocompatibility, non-toxicity, low extraction cost, and ease of gelation [75]. The polymer allows for a rapid, simple, and adjustable crosslinking process, with careful concentration considerations for optimal bioink viscosity. In wound healing application, alginate possesses high absorbency to maintain a moist environment and adsorb exudates [76]. Besides, alginate’s ionic nature enables the formation of biologically active polymer complexes, particularly with chitosan, facilitating effective drug delivery [77]. However, these notable advantages are accompanied by certain limitations. These include post-printing morphological instability and relatively soft mechanical properties of scaffolds [75]. Despite these challenges, the unique properties of alginate underscore its significant potential and ongoing importance in the field of bioprinting for wound healing.

#### Collagen

Collagen is an abundant natural protein in the body, composed of self-aggregated peptide chains bound by hydrogen and covalent bonds [78, 79]. As a biomaterial, exhibits several notable advantages relevant to wound healing applications. Firstly, collagen dressings possess high water absorbency, effectively reducing the loss of proteins and electrolytes from wound exudates, thus mitigating the risk of wound dehydration [80]. Secondly, collagen is soluble in weakly acidic aqueous solutions and can undergo polymerization within 60 min at 37 °C and neutral pH, enabling the 3D printing of collagen scaffolds through precise control of pH and temperature [81]. Thirdly, collagen is a natural receptor for cellular adhesion, promoting cell attachment. However, a drawback of collagen materials is the lower fidelity in maintaining shape [82].

#### Chitosan

Chitosan, derived from shellfish, exhibits high biocompatibility and biodegradability, accompanied by intriguing antimicrobial properties [83]. As a wound dressing, chitosan enhances the network structure of wound tissue, promoting collagen synthesis and reinforcing tensile strength [84]. Modified chitosan hydrogels have demonstrated efficacy in wound hemostasis [85]. Besides, the degradation products of chitosan are absorbable by epidermal cells, sustaining their growth and proliferation [86]. Chitosan has been observed to activate macrophages, inducing the release of cytokines such as transforming growth factor (TGF), platelet-derived growth factor (PDGF), and interleukin-1 (IL-1) [87]. Additionally, chitosan can effectively crosslink with other polyelectrolytes, particularly anionic polymers like alginate or hyaluronic acid, making it suitable for 3D bioprinting applications [88]. However, its relatively low mechanical properties and long gelation time pose a potential limitation in tissue engineering [89].

#### Cellulose

Cellulose, characterized by diverse and tunable mechanical, structural, chemical, and physical properties, proves versatile for the wide-ranging application in the fabrication of biomaterials for wound healing [90, 91]. It is easily obtainable and cost-effective [92]. Besides, cellulose boasts high biocompatibility, biodegradability, and moisturizing attributes, contributing to skin regeneration, drug delivery, and wound healing [93]. Moreover, cellulose demonstrates the ability to release various growth factors at the injury site, facilitating the migration and proliferation of dermal fibroblasts while inhibiting bacterial proliferation and expediting wound healing [92].

#### Silk fibroin

Silk fibroin protein is derived from silk produced by silkworms and spiders, and has been investigated for wound healing applications [94]. It exhibits excellent biocompatibility and biodegradability [95]. Silk fibroin can promote wound cell growth, differentiation, and extracellular matrix regeneration [96]. It provides a balance of tensile strength, modulus, and elongation for wound dressings, mimicking the mechanical behavior of normal tissues and facilitating tissue regeneration [97]. However, its drawback lies in its low viscosity, posing challenges in the 3D printing manufacturing of bioinks. One solution is the development of composite hydrogels by combining silk fibroin with SA for application in bioprinting [98].

#### Gelatin

Gelatin is obtained from partial hydrolysis of collagen. It exhibits excellent biocompatibility, non-immunogenicity, and cell affinity, and degrades completely in vivo, showcasing substantial potential as an ideal bioink [99]. For wound healing, gelatin can provide cell anchoring sites to promote cell adhesion [100]. Notably, it demonstrates a thermal response, undergoing crosslinking at low temperatures, and de-crosslinking as the temperature rises to physiological or higher levels [99]. Exploiting this property facilitates convenient adjustment of crosslinking during the bioprinting process, aiding in adapting and maintaining the shape of bio-printed objects, such as conforming to wound shapes and promoting wound contraction [101, 102]. Furthermore, introducing chemical substances like metal ions can enhance the printability and stability of gelatin [103].

#### Hyaluronic acid

Hyaluronic acid, the most common component in the ECM, is ubiquitously distributed in the human body and plays a pivotal role in various biological processes. In wound healing, it is intricately involved in inflammation, tissue recovery, regeneration, maintaining ECM integrity, and regulating cellular functions. As a signaling molecule, HA can promote cell adhesion, migration, and proliferation [104]. Additionally, its water-soluble nature and high viscosity make it a suitable material for bioprinting, often employed to modulate the viscosity of other biomaterial solutions [105, 106]. However, native HA is gelatinous with poor mechanical properties and rapid degradation. To address these limitations, modification of HA with methacrylate (MA) is commonly undertaken, enabling photo crosslinking under UV irradiation to enhance its mechanical performance [107].

#### Decellularized extracellular matrix

Decellularized extracellular matrix is a naturally derived biomaterial obtained through the decellularization of native tissues, preserving the structural and functional features of the ECM, making it an excellent material for bioprinting applications [108]. It retains bioactive components, such as collagen, glycosaminoglycans, and growth factors, which facilitate cellular processes like adhesion and proliferation to promote wound healing [109]. For instance, 3D-printed skin using dECM from porcine sources exhibits a structure similar to natural skin, and was found to promote epithelialization in chronic wound healing applications [110]. However, it faces challenges including the potential presence of residual pathogens and immunogenic reactions [111].

## Synthesis methods of EVs combined with hydrogels

**Fig. 2:**
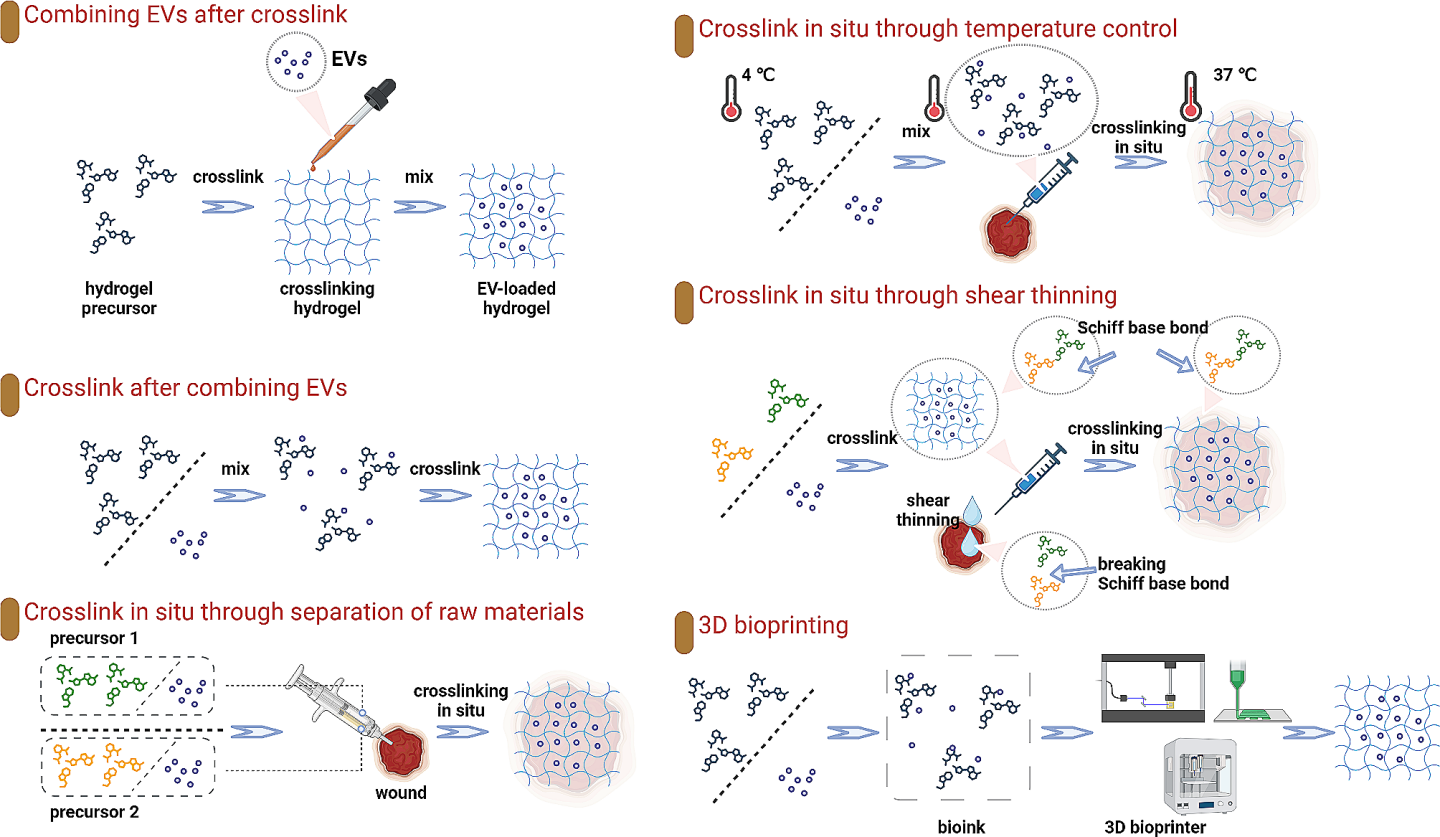
The synthesis methods of EVs combined with hydrogels for wound healing. The synthetic approaches for EV-loaded hydrogels can be categorized as follows: (1) Combining EVs after crosslink: sequential crosslinking followed by EV absorption; (2) Crosslink after combining EVs: pre-mixing EVs with hydrogel precursor and subsequent crosslinking; (3) Crosslink in situ through separation of raw materials: simultaneous injection of cross-linkable precursors mixed with EVs at the wound site for in-situ gelation; (4) Crosslink in situ through temperature control: injection of EVs mixed with thermosensitive hydrogel precursors, achieving in-situ gelation through temperature changes; (5) Crosslink in situ through shear thinning: pre-crosslinking of EVs with shear-thinning hydrogels, leading to hydrogel liquefaction through injection-induced shear forces to adapt to wound contours and subsequent in-situ gelation; (6) 3D bioprinting: formation of a bio-ink by combining EVs and hydrogel precursors, followed by 3D printing technology for composite hydrogel preparation

To adapt to the conditions and environments of wounds, achieve sustained release of EVs, or meet specific therapeutic demands, many studies have been conducted to combine EVs and hydrogels for better wound-healing effects. Hydrogels are formed after the precursor materials are crosslinked under specific conditions or through the action of a crosslinking agent. The manner in which hydrogels are crosslinked to form scaffolds, as well as the timing of EV integration with precursor materials or mature hydrogels, determines the efficacy of the binding methods. This part includes recent explorations of EV-loaded hydrogels in wound treatment, and the efforts in the development of 3D bioprinting, tailored to specific therapeutic purposes. (Fig. 2) And Table 3 summaries the recent studies including the synthesis methods of EVs combined with hydrogels for wound healing.

**Table 3:** Summary synthesis methods of EVs combined with hydrogels for wound healing

Synthesis method	Sources of EV	Hydrogel material	Detail method	Result	References
Combining EVs after crosslink	PRP	Chitosan/silk hydrogel	EVs absorbed by cross-linked hydrogels	Promote diabetic wound healing, enhance collagen deposition and accelerate angiogenesis	[59]
	GMSC	Chitosan/silk hydrogel	EVs absorbed by cross-linked hydrogels	Promote diabetic wound healing, enhance collagen deposition, accelerate angiogenesis and nerve regeneration	[54]
	Periosteum cells	NAGA/GelMA/Laponite/glycerol hydrogel	EVs absorbed by cross-linked hydrogels	Promote FB and HUVEC viability, proliferation, migration, tube formation and the expression of FGF-1; promote diabetic wound healing, enhance collagen deposition, accelerate angiogenesis	[112]
Crosslink by agents after combining EVs	HUVEC	GelMA	The mixture of GelMA, photoinitiator 2959 and HUVEC-EVs crosslinked under UV light	Promote FB and HaCaT viability, proliferation and migration; promote wound healing, enhance collagen deposition, accelerate angiogenesis	[50]
	ADSC	alginate hydrogel	The mixture of alginate and ADSC-EVs crosslinked by adding calcium chloride (CaCL2) solution	Promote HUVEC viability, proliferation and migration; promote wound healing, enhance collagen deposition, accelerate angiogenesis	[66]
	ESC	GelMA	The mixture of GelMA, LAP photoinitiator and ESC-EVs crosslinked under UV light	Promote HUVEC viability, proliferation, migration and tube formation; promote diabetic wound healing, enhance collagen deposition, accelerate angiogenesis	[55]
Crosslink without agents after combining EVs	human endometrial stem cell (hEnSCs)	chitosan/glycerol hydrogel	The stable hydrogel construct was obtained through electrostatic interaction between positive NH2 charges of Ch and negative OH charge of glycerol as well as hydrogen-bonding interactions between the Ch chains.	Promote FB viability, proliferation and migration; promote wound healing, enhance collagen deposition, accelerate angiogenesis	[113]
	Synovium-derived stem cells	Chitosan hydrogel	The hydrogel mixture with SMSC-EVs was placed at -20 °C for 2 h, and then NaOH solution was added and the mixture was kept at 4 °C for 4 h	Promote FB and HMEC-1 viability, proliferation, migration and tube formation; promote diabetic wound healing, enhance collagen deposition, accelerate angiogenesis	[57]
	royal jelly	Type I collagen hydrogel	The hydrogel was mixed with royal jelly derived EVs at 4 °C and polymerized at 37 °C	Promote FB viability, proliferation and migration; form antibacterial biomembrane	[60]
Crosslink in situ through shear thinning	ADSC	FHE hydrogel (F127/OHA-EPL)	The hydrogel was injected into wounds after the solid-liquid transition under injection pressure and quickly gelated in situ	Promote HUVEC viability, proliferation, migration and tube formation; promote diabetic wound healing, enhance collagen deposition, accelerate angiogenesis	[37]
	ADSC	FEP scaffold (F127-PEI/APu)	The hydrogel was injected into wounds after the solid-liquid transition under injection pressure and quickly gelated in situ	Promote HUVEC viability, proliferation, migration and tube formation; promote diabetic wound healing, enhance collagen deposition, accelerate angiogenesis and cell proliferation in wounds	[114]
	PMSCs	methylcellulose-chitosan hydrogel	The hydrogel was injected into wounds after the solid-liquid transition under injection pressure and quickly gelated in situ	Promote diabetic wound healing, enhance collagen deposition, accelerate angiogenesis and regulate the expression of Bcl-2, Bax and VEGF	[115]
Crosslink in situ through temperature control	hUCMSC	PF-127 hydrogel	The hydrogel mixture stored at 4 °C and the gel transition happened at 37 ℃ on wounds	Promote HUVEC viability, proliferation and migration; promote diabetic wound healing, enhance collagen deposition, accelerate angiogenesis and cell proliferation in wounds, and regulate the expression of VEGF and tgf-β	[73]
	ADSC	PF-127 hydrogel	The hydrogel mixture stored at 4 °C and the gel transition happened at 37 ℃ on wounds	Promote wound healing, enhance collagen deposition, accelerate angiogenesis and cell proliferation in wounds, and regulate wound inflammation	[116]
	M2 macrophage	HAh and Haaq hydrogel	Both hydrogel precursors were injected in situ on the wounds and the gel transition happened by an efficient Schiff base reaction between the hydrazide moieties of HAh and the aldehyde moieties of HAaq	Promote FB and HUVEC viability, proliferation and tube formation; promote diabetic wound healing, enhance collagen deposition, accelerate angiogenesis and reduce wound ROS level	[45]
3D bioprinting	THP-1-derived activated macrophages (MΦ)	LVG-RGD/gelatin hydrogel	3D bioprinting was performed using the freeform reversible embedding of suspended hydrogel (FRESH) approach	Promoted NRCM viability, metabolic activity, and reduced their apoptosis	[117]
	Lyosecretome	SA-SF hydrogel	Printability and shape fidelity of the SA-SF hydrogel were assessed using CELLINK INKREDIBLE+, an extrusion-based 3D bioprinter	Realize controllable EV-release kinetics with better EV-controlled-release effect	[118]
	Lyosecretome	PCL/alginate hydrogel	PCL scaffolds were 3D-printed with the Cellink INKREDIBLE+, a pneumatic extrusion-based 3D bioprinter	Realize controllable EV-release kinetics with better EV-controlled-release effect	[72]

### Combining EVs after crosslink

This method is also called the “breathing” method. The raw materials of the hydrogels are first crosslinked, and then EVs are introduced by removing the water in the pores of the hydrogels and immersing them in the EV solution so that EVs are composited into the hydrogels during the swelling procedure. This method involves simple steps. However, the disadvantage is that the pore size of the hydrogels needs to be larger than the size of EVs to absorb EVs into the hydrogels, whereas an excessive pore size will lead to the acute release of EVs and lose the capacity for sustained release [119]. This leads to the requirement for a rigorous pre-design of composite hydrogels.

Han et al. first used ultraviolet radiation to crosslink raw materials to obtain a NAGA/GelMA/liponite/glycerol hydrogel and then dropped a solution of EVs from the periosteum (P-EVs) into the hydrogel to form a composite hydrogel (N-acryloyl glycinamide, NAGA). The hydrogel significantly promoted the viability and migration of FBs, tube formation of HUVECs, and wound healing in diabetic rats [112]. Tang et al. synthesized a composite hydrogel scaffold based on GelMA and hUCMSC-EVs. GelMA hydrogel was prepared through crosslinking, followed by the dropwise addition of the EV solution onto the hydrogel to facilitate absorption. This hydrogel demonstrated the capacity to modulate wound inflammation and promote the healing of diabetic rat wounds [120].

### Crosslink after combining EVs

In this method, the EV solution is initially combined with hydrogel precursor materials and then crosslinked using a crosslinking agent or altering the physical conditions. This method allows for the preparation of composite hydrogels with minimized EV loss and enables precise control over the total amount and proportion of EVs and hydrogel materials. Furthermore, it offers the flexibility to design smaller pore sizes, facilitating increased EV encapsulation. As the hydrogels undergo swelling and degradation, a gradual release of EVs occurs. However, a potential drawback is that crosslinking conditions, such as ultraviolet radiation or the use of certain crosslinking agents, may impact EV activity and require careful consideration.

#### Crosslink by agents after combining EVs

First, an alternative method involves using the crosslinking agents to crosslink the hydrogel after combining EVs. The hydrogel is crosslinked by adding crosslinking agents to a mixed solution of EVs and the raw materials of the hydrogels. GelMA is a commonly used porous hydrogel that can be crosslinked under ultraviolet radiation after adding a crosslinking agent, such as a lithium acyl phosphinate photoinitiator (LAP) [121]. Wang et al. used GelMA to mix EVs derived from ESCs, and the solution formed cross-linked hydrogels upon exposure to ultraviolet light for 10 s after optimizing the concentration of GelMA, and the activity of EVs was not affected [55]. Xu et al. developed a hydrogel based on recombinant human collagen (rhCol) III. By adding a transglutaminase (TGase) crosslinker to the mixture of hUCMSC-EVs and rhCol III, the mixture was crosslinked, and TGase tightly promoted EVs adhesion to the collagen surface, thus extending the sustained release time of EVs [122].

#### Crosslink without agents after combining EVs

In crosslinking without agents, specific physical conditions, such as low temperatures and changes in pH, are used to crosslink mixed solutions of EVs and hydrogel precursors. For example, Kwak et al. mixed the separate precursor components of a degradable PEG hydrogel with EVs derived from M2 macrophages and controlled the gelation time by controlling the pH of the solution [58]. Ramírez et al. used a type I collagen hydrogel mixed with EVs from royal jelly for wound healing. The gel was liquid at 4 ℃ and the gel transition happened at 37 ℃; thus, the timing of gelation could be easily controlled by changing the temperature [60].

### Crosslink in situ

Many applications of this method are similar to the method of “crosslinking after combining EVs.” However, in this method, the hydrogels have unique properties such as injectability or heat sensitivity, and crosslinking and gelation occur in situ to better adapt to the shapes of wounds or meet different demands for wound healing.

#### Crosslink in situ through separation of raw materials

To conduct a crosslink in situ while maintaining the separation of raw materials, two or more precursor materials of the hydrogels were first mixed with EVs separately, and then mixed solutions were injected in situ on the wounds at the same time so that crosslinking and gelation occurred automatically on the wound surface to adapt to the shapes of the wounds. Xiong et al. used this method to design multifunctional hydrogels. The two precursor materials, hydrazide grafted HA (HAh) and aldehyde and quaternary ammonium grafted HA (HAaq), were mixed with the EVs solution and then injected in situ through a dual-chamber syringe simultaneously. The hydrogel was formed through cross-linking of the reversible Schiff base bonds of HAh and HAaq [45].

#### Crosslink in situ through temperature control

To conduct a crosslink in situ through temperature control, temperature-sensitive liquid hydrogels mixed with EV solutions are applied to the wound surface. This has the advantages of convenient administration and automatic gelation. For example, the Pluronic F127 hydrogel used by Yang et al. exhibits temperature sensitivity. After optimizing the concentration parameters, the solution mixed with hUCMSC-EVs was applied to the wound in liquid form, and the hydrogel was formed at the wound temperature of 37 ℃ in approximately one min [73].

#### Crosslink in situ through shear thinning

Some hydrogels possess shear-thinning properties, enabling in situ crosslinking through shear forces. The hydrogel networks’ structure is stabilized through noncovalent interactions when no force is applied to the hydrogels. However, the interaction between networks is reversible and dynamic, and the crosslinking networks can dissociate under shear force, leading to hydrogel liquefaction while returning to the solid state after the relief of external stress [123]. Therefore, this type of hydrogel can be prepared in advance in vitro, injected into wounds after the solid-liquid transition under injection pressure, and quickly gelated in situ.

For example, Wang et al. designed a bioactive antibacterial adaptive ultraviolet shielding nano dressing consisting of a Pluronic F127 hydrogel and grafted polyethylene (PEI) and aldehyde pullulan (APu). Due to the large number of amino groups from PEI and aldehydes from APu, the hydrogel was endowed with a shear-thinning property by hydrogen bonding and Schiff base reactions with the wounds. Therefore, the hydrogel mixed with ADSC-EVs could be injected in situ through a syringe and was well-adapted to the size of the wound to provide antibacterial and ultraviolet shielding properties [114]. Wang et al. used a methylcellulose-chitosan hydrogel consisting of two raw materials: aldehyde-modified methylcellulose (MC-CHO) and chitosan-grafted polyethylene glycol (CS-g-PEG). The Schiff base reactions of the aldehyde groups in MC-CHO and the amino groups in the CS chains endowed the hydrogel with shear-thinning characteristics [115].

### 3D bioprinting

3D bioprinting techniques include laser-assisted, inkjet, and extrusion-based printing. These techniques involve the printing of biomaterials, often combined with cells or bioactive substances, in the form of bioinks. The technology enables the creation of multi-layered tissue structures that mimic normal human tissue, and allows for the digital customization and automated printing of personalized artificial scaffolds to meet specific individual requirements [27].

3D bioprinting technology, compared to traditional hydrogel manufacturing methods, allows for the fabrication of more complex tissue-mimicking bio-scaffolds with tunable properties [124]. Li et al. demonstrated the precision engineering of a composite hydrogel scaffold loaded with ADSC-EVs for cartilage regeneration. The raw materials included GelMA, dopamine-conjugated HA (HA-DA), and OHA, forming a unique spatial hydrogel scaffold with a double network that provided a tissue-specific microenvironment, and the composition of various hydrogels was optimized to achieve optimal mechanical properties. 3D bioprinting technology emerged as an ideal strategy for realizing this complex design [125].

In comparison to traditional methods, 3D bioprinting also enhances the EV release capability of scaffolds. For instance, Elia et al. utilized Lyosecretome, a freeze-dried formulation containing EVs from MSCs, added to SA-SF hybrid hydrogel ink, and controlled scaffold design parameters were employed to achieve regulated EV release kinetics [118]. Lyosecretome was also incorporated into PCL/alginate based composite hydrogels. The study revealed that, compared to traditional scaffolds that absorbed EVs via diffusion, 3D-bioprinted scaffolds using a mixed bioink exhibited superior EV release effects [72]. The versatility of 3D bioprinting technology provides a promising avenue for optimizing the design and performance of composite hydrogel scaffolds, facilitating controlled EV delivery for enhanced tissue regeneration applications.

In the context of wound healing, 3D bioprinting offers high precision in fabricating hydrogel materials tailored to the shape of the wound. For instance, Huang et al. developed a tissue-friendly, high-efficiency, and accurate 3D-printed all-peptide hydrogel platform based on the thiol-ene click reaction. The printing accuracy and resolution reached below 1 mm, allowing for rapid construction within minutes [126]. Besides, the support provided by 3D bioprinting for intricate scaffolds allows for the incorporation of various additional features into hydrogel dressings. This enables the display of excellent mechanical properties and additional wound healing capabilities. For example, the addition of conductive materials can promote wound healing by modulating cell proliferation, migration, and local inflammation levels [127]. Moreover, 3D printing enables the customization of scaffolds with tissue-specificity, simulating normal tissue conditions for enhanced therapeutic effects.For example, Byoung et al. used skin derived extracellular matrix (S-dECM) bioink for 3D bioprinting of skins. The S-dECM bioink retained major ECM compositions of skin, including viable growth factors and cytokines, and helped to form a microenvironment that promoted the proliferation and differentiation of cells seeded on the scaffolds and facilitated wound healing [128].

Building upon the achievements mentioned above, the therapeutic application of 3D bioprinting on EV-loaded hydrogels in wound healing is garnering increased attention. Zhong et al. attempted to construct a three-dimensional scaffold hydrogel loaded with ADSC-EVs using Gel/dECM/Qcs (quaternized chitosan, Qcs), and employed the positive charge of quaternary ammonium salts to anchor EVs. They confirmed the excellent mechanical properties, biocompatibility, and antimicrobial capacity of 3D hydrogel scaffolds for wound healing [129]. Similarly, Louis et al. developed a GelMA composite hydrogel loaded with MSC-EVs, showcasing significant promotion of angiogenesis and tissue repair and regeneration [130]. Letizia et al. engineered a 3D patch using MeHA loaded with MSC-EVs, demonstrating the ability to enhance wound cell proliferation, migration, and vascularization. Su et al. utilized PCL as an electrospinning material to construct a three-dimensional scaffold structure. They immobilized PEI onto the scaffold’s porous structure, facilitating the adsorption of negatively charged MSC-EVs. Animal experimental results indicated that this composite hydrogel could modulate wound inflammation levels to promote wound healing [131]. In conclusion, the combination of EV-loaded hydrogels with 3D bioprinting holds tremendous potential in the field of wound healing.

## Effects of EV-loaded hydrogels in wounds

### Promoting the proliferation and migration of wound cells

EV-loaded hydrogels facilitate wound healing and promote the biological functions of wound cells, such as FBs, keratinocytes, and vascular endothelial cells. These cells play important roles in wound healing, and their impaired cell proliferation or function caused by unfavorable wound microenvironments may lead to prolonged wound healing or chronic wounds [132–135]. As described above, the use of EVs or hydrogels alone is conducive to promoting the biological functions of wound cells, and this effect is strengthened when EVs and hydrogels are combined.

### Regulating the collagen deposition and scar formation

During the proliferative phase of wound healing, actively proliferating FBs produce and promote collagen deposition. Collagen deposition is a necessary process for wound healing [136]. Therefore, an increase in collagen deposition represents better wound healing efficiency. Studies have shown that both EVs and hydrogels can promote collagen deposition in wounds and that the combination of both treatments enhances this effect. For example, Wang et al. used MSC-EV and GelMA-DOPA-EV hydrogels on diabetic wounds and found that collagen deposition in the GelMA-DOPA-EV and EVs groups increased similarly on day 7, whereas the GelMA-DOPA-EV group showed a more significant increase on day 14 [137].

However, excessive collagen deposition is considered to be a key factor in scar formation [136]. In the later stages of wound healing, controlling excessive FB activity and inhibiting collagen deposition can suppress scar formation [138]. Shen et al. designed a bilayer hydrogel in which BMSC-EVs in the lower layer were first released, promoting FB function and collagen deposition at the early stage of wound healing. In the later stage (7 days), the upper layer gradually released EVs with high miR-29b-3p expression and inhibited fibrosis-related gene expression and collagen deposition in the scar tissue, thereby reducing scar formation [139].

### Facilitating angiogenesis

Angiogenesis is essential for wound healing. Injured blood vessels first expand to increase the blood supply around the wound, which contributes to the absorption of nutrients and growth factors as well as the migration and settlement of vascular endothelial cells, keratinocytes, and FBs. Then, the secretion of growth factors such as TGF- β, ILs, and angiogenic factors promote further proliferation and sprouting of local blood vessels. EV-loaded hydrogels can promote wound angiogenesis by loading EVs with effective angiogenic abilities or by delivering drugs that can promote vascular regeneration. Hu et al. engineered GelMA-based hydrogels loaded EVs derived from hypoxia-treated ADSCs, revealing enhanced therapeutic effects in wound healing compared to normal EVs. The delivery of circ-Snhg11 activated the miR-144-3p/NFE2L2/HIF1α pathway, enhancing vascular endothelial cell survival and function and promoting wound healing [61].

### Regulating inflammation

Macrophages are primary cells that regulate inflammation during wound recovery, playing a crucial role in wound healing. In the early stages, macrophage activation is induced, and a series of inflammatory responses occur. Activated macrophages can polarize into the M1 and M2 phenotypes. M1 phenotype macrophages mainly play a pro-inflammatory role, producing pro-inflammatory cytokines such as tumor necrosis factor α (TNF-α) and IL-6, etc. Moreover, they have a strong phagocytic ability to clear pathogens or cell debris from wounds [140]. M2 macrophages usually have anti-inflammatory effects and can secrete anti-inflammatory cytokines, such as IL-10, and promote wound remodeling and healing [141]. The transition from M1 to M2 macrophages is crucial for efficient wound recovery, with prolonged or stalled transitions contributing to delayed healing and chronicity [5, 142]. Therefore, regulating macrophage polarization is an important strategy to accelerate wound healing, and some EV-loaded composite hydrogels effectively promote M2 macrophage polarization. Kwak et al. used EVs derived from M2 macrophages, which can induce polarization from M1 to M2, thereby controlling wound inflammation and promoting the transition to the proliferative phase of wound healing. A composite microneedle hydrogel with MSC-derived EVs was used by Zhang et al. to regulate macrophage polarization in vitro. In vivo, the content of TNF-α significantly decreased after hydrogels were used in diabetic wounds [70].

### Promoting the regeneration of hair follicles, nerves and other accessory organs

The effective healing of wounds involves not only the closure of the wound area, but also the regeneration of its full functions, which are closely linked to accessory organs such as nerves and hair follicles. For example, scar-formed wound healing results in the loss of normal skin functions, with only 80% of the skin’s strength [143] and no hair follicles or other accessory organs [136]. Hair follicles are complex appendages with complex structures and functions that are difficult to regenerate. Hair follicles may differentiate from epidermal cells, such as basal keratinocytes, during the process of wound healing [144]. Despite these difficulties, some studies have used composite hydrogels with EVs on wounds to achieve hair follicle regeneration. For example, a Pluronic F127 hydrogel combined with hUCMSC-EVs designed by Yang et al. was applied to diabetic wounds, and slices of the wound tissues at 14 days showed hair follicle regeneration, but not in the group using hydrogels or EVs alone. This may be due to the Wnt, Bmp, Notch, and Sonic hedgehog (SHH) pathways, which are involved in hair follicle regeneration and wound healing [145]. Nerves in the skin tissue are part of the peripheral nervous system, and many wounds are accompanied by nerve damage [146]. Research has confirmed that some EVs can promote nerve regeneration [147], and additional drugs can be added to EV-loaded hydrogels to enhance this effect. Qian et al. designed a composite hydrogel combining silver nanoparticles (AgNPs) and hUCMSC-EVs, which enhanced the effect of nerve regeneration by EVs [148]. Research has demonstrated that inflammatory cells and factors caused by wound infections may inhibit the repair of nerves [149], while AgNPs endow hydrogels with antibacterial properties to suppress the inflammatory environment and promote nerve regeneration.

## Conclusions, challenges and prospects

Recently, EVs have received considerable attention in the exploration of new therapeutic methods for wound healing. Hydrogels have demonstrated the ability to compensate for the shortcomings of EVs when applied to wounds. Therefore, the development of composite materials for EVs and hydrogels to promote wound regeneration is crucial. Subsequently, diverse combinations of hydrogels and EVs have been developed to endow composite hydrogels with extended sustained-release ability and excellent pro-healing properties. The 3D bioprinting of EV-loaded hydrogels will provide more attractive platforms for basic and translational medical applications.

However, EV-loaded hydrogels still face some potential challenges: (1) EVs: establishing a standardization of production, extraction, and storage methods for EVs to provide stable and reliable therapeutic EVs; (2) hydrogels: improving hydrogels with better characteristics to adapt to more clinical scenarios; and (3) synthesis methods: optimizing manufacturing strategies to reduce adverse effects on EVs and further improve the performance of the composite hydrogels.

Standardized processes must be established for the use of EVs in wound treatment. When used as a therapeutic drug delivery system, different cultivation and extraction methods may influence the viability of EVs or introduce foreign pollutants into their products. In addition, different characterization and counting methods affect the quality control of EVs [150]. Although research on EVs has made progress recently, a comprehensive and detailed understanding of EV functions and mechanisms, and the composition and characterization of EV subgroups is still lacking. Therefore, it is necessary to develop standard protocols for the source, collection, processing, extraction, characterization, and data analysis of EVs to establish standardized processes for EV production [151]. Moreover, there is currently a lack of reported preservation methods for EVs in composite hydrogels. It is generally believed that EVs need to be stored at -80 degrees to maintain their activity, and repeated freeze-thaw cycles can lead to a loss of their activity [152]. In current research, composite hydrogels are typically prepared and immediately used, with a lack of relevant experiments on their storage.

Another opportunity is to explore hydrogels with improved properties. Natural and synthetic hydrogels have their own advantages; however, their disadvantages cannot be ignored. For example, natural hydrogels lack stability, mechanical properties, and tissue adhesion properties [153], while synthetic hydrogels have the disadvantages of relatively poor biocompatibility [64], toxic precursors, and degradation products [154] and require further careful design in clinical applications. Many studies have combined natural (or modified natural) hydrogels with synthetic hydrogels to compensate for these drawbacks and reinforce the strengths of each method. For example, in the GelMA-SFMA composite hydrogel designed by Zhu et al., the good elasticity of SFMA compensates for the low mechanical modulus and limited pore size and porosity of GelMA [155], whereas the interpenetrating polymer network formed by the combination of GelMA and SFMA further strengthens the mechanical properties of the hydrogels [67]. Besides, nano materials can also be added to the hydrogels, such as organic nanomaterials to improve the mechanical strength and conductivity, and metal nano materials to increase their responsiveness to magnetism and other factors [156].

Moreover, it is very important to further optimize the manufacturing strategies of EV-loaded hydrogels to minimize the adverse effects on EVs. The crosslinking agents used in the general manufacturing methods may cause damage to EVs. High ultraviolet light intensity or high temperature in 3D bioprinting techniques such as stereolithography or fused deposition modeling may lead to the inactivation of EVs, while bioprinting based on extrusion is considered to remain EV viability mostly [156, 157]. However, as research progresses, the shear stress in extrusion based bioprinting was found to cause cell death in the hydrogels [158]. After improving the manufacturing strategies, shear-thinning hydrogel was found to reduce shear stress during the extrusion process and reduce the impact on biomolecules [159]. Therefore, while improving manufacturing strategies to produce the composite hydrogels with excellent performance, the impact on scaffolds’ bioactive components also needs to be considered. It is essential to optimize manufacturing methods to attain the ideal balance between high performance and maintenance of bioactive molecular activity. In recent studies, machine learning has been employed to construct and analyze physical models, proposing more innovative printing approaches and maintaining the biocompatibility and structural integrity in 3D-bioprinting processes [160, 161]. This may contribute to reducing the loss of bioactive components and enhancing printing performance and the biological effects of 3D scaffolds in the future.

In conclusion, EV-loaded composite hydrogels represent an effective strategy for wound healing and tissue regeneration with numerous advantages. In the future, a deeper understanding of the characteristics of EVs and hydrogels will aid in selecting raw materials with different properties to meet the personalized treatment needs of different patients. Additionally, with the continuous development of technology, the integration of high-precision printing techniques will further enhance the performance of EV-loaded hydrogels and advance their clinical applications. This will provide new therapeutic avenues in the fields of tissue repair and regenerative medicine.

## Data Availability

The datasets generated during and/or analyzed during the current study are available from the corresponding author on reasonable request.

## Abbreviations

ADSCs: Adipose-derived stem cells
APu: Aldehyde pullulan
BMSCs: Bone marrow stem cells
CS: Chitosan
CS-g-PEG: Chitosan-grafted polyethylene glycol
DPSCs: Dental pulp stem cells
ECM: Extracellular matrix
EPCs: Epidermal stem cells
EPL: Poly-ε-L-lysine
EVs: Extracellular vesicles
FBs: Fibroblasts
GelMA: Gelatin methacryloyl
GMA: Glycidyl methacrylate
GMSCs: Gingival stem cells
HA: Hyaluronic acid
HAaq: Aldehyde and quaternary ammonium grafted HA
HA-DA: Dopamine-conjugated HA
HAh: Hydrazide grafted HA
hUCB-MNCs: Human umbilical cord blood mononuclear cells
hUCMSCs: Human umbilical cord mesenchymal cells
HUVECs: Human umbilical vein endothelial cells
ILs: Interleukins
LAP: Lithium acyl phosphinate photoinitiator
MC-CHO: Aldehyde-modified methylcellulose
MeHA: Methacrylated hyaluronic acid
MSCs: Mesenchymal stem cells
NAGA: N-acryloyl glycinamide
NRCMs: Neonatal rat cardiomyocytes
OHA: Oxidative hyaluronic acid
PCL: Poly(ε-caprolactone)
PDGF: Platelet-derived growth factor
PEG: Polyethylene glycol
PEI: Polyethylene / polyethylenimine
P-EVs: EVs from the periosteum
PMSCs: Placental mesenchymal stem cells
PRP: Platelet-rich plasma
PU: Polyurethane
PVA: Polyvinyl alcohol
RGD: Arginine-glycine-aspartate
rhCol: Recombinant human collagen
ROS: Reactive oxygen species
SA: Sodium alginate
S-dECM: Skin derived extracellular matrix
SF: Silk fibroin
SHH pathways: Sonic hedgehog pathways
TGase: Transglutaminase
TGF-β: Transforming growth factorβ
TNF-α: Tumor necrosis factorα
VEGF: Vascular endothelial growth factor

## References

[1] Canedo-Dorantes L, Canedo-Ayala M. Skin Acute Wound Healing: A Comprehensive Review. *Int J Inflam* 2019, 2019:3706315. 10.1155/2019/3706315 PMC6582859 31275545

[2] Raziyeva K, Kim Y, Zharkinbekov Z, Kassymbek K, Jimi S, Saparov A. Immunology of Acute and Chronic Wound Healing. *Biomolecules* 2021, 11. 10.3390/biom11050700 PMC8150999 34066746

[3] Kuraitis D, Rosenthal N, Boh E, McBurney E (2022). Macrophages in dermatology: pathogenic roles and targeted therapeutics. Arch Dermatol Res.

[4] Marshall CD, Hu MS, Leavitt T, Barnes LA, Lorenz HP, Longaker MT (2018). Cutaneous scarring: Basic Science, current treatments, and future directions. Adv Wound Care (New Rochelle).

[5] Zhao R, Liang H, Clarke E, Jackson C, Xue M. Inflammation in chronic wounds. Int J Mol Sci 2016, 17. 10.3390/ijms17122085 PMC5187885 27973441

[6] Han G, Ceilley R (2017). Chronic Wound Healing: a review of current management and treatments. Adv Ther.

[7] Heras KL, Igartua M, Santos-Vizcaino E, Hernandez RM (2020). Chronic wounds: current status, available strategies and emerging therapeutic solutions. J Controlled Release.

[8] Shi Y, Wang S, Zhang W, Zhu Y, Fan Z, Huang Y, Li F, Yang R (2022). Bone marrow mesenchymal stem cells facilitate diabetic wound healing through the restoration of epidermal cell autophagy via the HIF-1α/TGF-β1/SMAD pathway. Stem Cell Res Ther.

[9] Nie C, Yang D, Xu J, Si Z, Jin X, Zhang J (2011). Locally administered adipose-derived stem cells accelerate wound healing through differentiation and vasculogenesis. Cell Transpl.

[10] Noiseux N, Gnecchi M, Lopez-Ilasaca M, Zhang L, Solomon SD, Deb A, Dzau VJ, Pratt RE (2006). Mesenchymal stem cells overexpressing akt dramatically repair infarcted myocardium and improve cardiac function despite infrequent cellular fusion or differentiation. Mol Ther.

[11] Basu J, Ludlow JW (2014). Cell-based therapeutic products: potency assay development and application. Regen Med.

[12] Hade MD, Suire CN, Mossell J, Suo Z (2022). Extracellular vesicles: emerging frontiers in wound healing. Med Res Rev.

[13] Niel G, D’Angelo G, Raposo G (2018). Shedding light on the cell biology of extracellular vesicles. Nat Rev Mol Cell Biol.

[14] Zheng M, Huang M, Ma X, Chen H, Gao X (2019). Harnessing exosomes for the development of Brain Drug Delivery systems. Bioconjug Chem.

[15] Tian T, Zhu YL, Zhou YY, Liang GF, Wang YY, Hu FH, Xiao ZD (2014). Exosome uptake through clathrin-mediated endocytosis and macropinocytosis and mediating miR-21 delivery. J Biol Chem.

[16] Joshi BS, Beer MA, Giepmans BNG, Zuhorn IS (2020). Endocytosis of Extracellular vesicles and release of their Cargo from endosomes. ACS Nano.

[17] Amariglio N, Hirshberg A, Scheithauer BW, Cohen Y, Loewenthal R, Trakhtenbrot L, Paz N, Koren-Michowitz M, Waldman D, Leider-Trejo L (2009). Donor-derived brain tumor following neural stem cell transplantation in an ataxia telangiectasia patient. PLoS Med.

[18] Zhang J, Guan J, Niu X, Hu G, Guo S, Li Q, Xie Z, Zhang C, Wang Y (2015). Exosomes released from human induced pluripotent stem cells-derived MSCs facilitate cutaneous wound healing by promoting collagen synthesis and angiogenesis. J Transl Med.

[19] Zhang B, Yin Y, Lai RC, Tan SS, Choo ABH, Lim SK (2014). Mesenchymal stem cells secrete immunologically active exosomes. Stem Cells Dev.

[20] Li M, Fang F, Sun M, Zhang Y, Hu M, Zhang J (2022). Extracellular vesicles as bioactive nanotherapeutics: an emerging paradigm for regenerative medicine. Theranostics.

[21] Verweij FJ, Balaj L, Boulanger CM, Carter DRF, Compeer EB, D’Angelo G, Andaloussi SE, Goetz JG, Gross JC, Hyenne V (2021). The power of imaging to understand extracellular vesicle biology in vivo. Nat Methods.

[22] Lázaro-Ibáñez E, Faruqu FN, Saleh AF, Silva AM, Wang JT-W, Rak J, Al-Jamal KT, Dekker N (2021). Selection of fluorescent, bioluminescent, and Radioactive tracers to accurately reflect extracellular vesicle biodistribution in vivo. ACS Nano.

[23] Trenkenschuh E, Richter M, Heinrich E, Koch M, Fuhrmann G, Friess W (2022). Enhancing the Stabilization Potential of Lyophilization for Extracellular vesicles. Adv Healthc Mater.

[24] Stephanopoulos N, Ortony JH, Stupp SI (2013). Self-assembly for the synthesis of functional biomaterials. Acta Mater.

[25] Annabi N, Tamayol A, Uquillas JA, Akbari M, Bertassoni LE, Cha C, Camci-Unal G, Dokmeci MR, Peppas NA, Khademhosseini A (2014). 25th anniversary article: Rational Design and Applications of hydrogels in Regenerative Medicine. Adv Mater.

[26] Safari B, Aghazadeh M, Davaran S, Roshangar L (2022). Exosome-loaded hydrogels: a new cell-free therapeutic approach for skin regeneration. Eur J Pharm Biopharm.

[27] Murphy SV, Coppi P, Atala A (2020). Opportunities and challenges of translational 3D bioprinting. Nat Biomed Eng.

[28] Yan W-C, Davoodi P, Vijayavenkataraman S, Tian Y, Ng WC, Fuh JYH, Robinson KS, Wang C-H (2018). 3D bioprinting of skin tissue: from pre-processing to final product evaluation. Adv Drug Deliv Rev.

[29] WICHTERLE O. LÍM D: Hydrophilic gels for Biological Use | Nature. Nature, 185:117–8.

[30] Li J, Mooney DJ (2016). Designing hydrogels for controlled drug delivery. Nat Rev Mater.

[31] Xu Y, Chen H, Fang Y, Wu J (2022). Hydrogel Combined with Phototherapy in Wound Healing. Adv Healthc Mater.

[32] Ma S, Hu H, Wu J, Li X, Ma X, Zhao Z, Liu Z, Wu C, Zhao B, Wang Y, Jing W (2022). Functional extracellular matrix hydrogel modified with MSC-derived small extracellular vesicles for chronic wound healing. Cell Prolif.

[33] Su J, Hu B-H, Lowe WL, Kaufman DB, Messersmith PB (2010). Anti-inflammatory peptide-functionalized hydrogels for insulin-secreting cell encapsulation. Biomaterials.

[34] Mardpour S, Ghanian MH, Sadeghi-abandansari H, Mardpour S, Nazari A, Shekari F, Baharvand H (2019). Hydrogel-mediated sustained systemic delivery of mesenchymal stem cell-derived extracellular vesicles improves hepatic regeneration in Chronic Liver failure. ACS Appl Mater Interfaces.

[35] Wu K, He C, Wu Y, Zhou X, Liu P, Tang W, Yu M, Tian W (2021). Preservation of small extracellular vesicle in gelatin methacryloyl hydrogel through reduced particles aggregation for therapeutic applications. IJN.

[36] Sun M, Li Q, Yu H, Cheng J, Wu N, Shi W, Zhao F, Shao Z, Meng Q, Chen H (2022). Cryo-self-assembled silk fibroin sponge as a biodegradable platform for enzyme-responsive delivery of exosomes. Bioactive Mater.

[37] W CWM, X T, Z X, L C, G W, X H (2019). Engineering Bioactive Self-Healing Antibacterial exosomes Hydrogel for promoting Chronic Diabetic Wound Healing and Complete skin regeneration. Theranostics.

[38] Han S, Yang H, Ni X, Deng Y, Li Z, Xing X, Du M (2023). Programmed release of vascular endothelial growth factor and exosome from injectable chitosan nanofibrous microsphere-based PLGA-PEG-PLGA hydrogel for enhanced bone regeneration. Int J Biol Macromol.

[39] Liu W, Gao R, Yang C, Feng Z, Ou-Yang W, Pan X, Huang P, Zhang C, Kong D, Wang W (2022). ECM-mimetic immunomodulatory hydrogel for methicillin-resistant Staphylococcus aureus-infected chronic skin wound healing. Sci Adv.

[40] Liu K, Chen C, Zhang H, Chen Y, Zhou S (2019). Adipose stem cell-derived exosomes in combination with hyaluronic acid accelerate wound healing through enhancing re-epithelialization and vascularization. Br J Dermatol.

[41] Zhang Y, Li M, Wang Y, Han F, Shen K, Luo L, Li Y, Jia Y, Zhang J, Cai W (2023). Exosome/metformin-loaded self-healing conductive hydrogel rescues microvascular dysfunction and promotes chronic diabetic wound healing by inhibiting mitochondrial fission. Bioact Mater.

[42] Yuan M, Liu K, Jiang T, Li S, Chen J, Wu Z, Li W, Tan R, Wei W, Yang X (2022). GelMA/PEGDA microneedles patch loaded with HUVECs-derived exosomes and Tazarotene promote diabetic wound healing. J Nanobiotechnol.

[43] Geng X, Qi Y, Liu X, Shi Y, Li H, Zhao L (2022). A multifunctional antibacterial and self-healing hydrogel laden with bone marrow mesenchymal stem cell-derived exosomes for accelerating diabetic wound healing. Biomaterials Adv.

[44] Xiang K, Chen J, Guo J, Li G, Kang Y, Wang C, Jiang T, Zhang M, Jiang G, Yuan M (2023). Multifunctional ADM hydrogel containing endothelial cell-exosomes for diabetic wound healing. Mater Today Bio.

[45] Xiong Y, Chen L, Liu P, Yu T, Lin C, Yan C, Hu Y, Zhou W, Sun Y, Panayi AC (2022). All-in-One: multifunctional hydrogel accelerates oxidative Diabetic Wound Healing through timed-release of exosome and fibroblast growth factor. Small.

[46] Shiekh PA, Singh A, Kumar A (2020). Exosome laden oxygen releasing antioxidant and antibacterial cryogel wound dressing OxOBand alleviate diabetic and infectious wound healing. Biomaterials.

[47] Pires AO, Mendes-Pinheiro B, Teixeira FG, Anjo SI, Ribeiro-Samy S, Gomes ED, Serra SC, Silva NA, Manadas B, Sousa N, Salgado AJ (2016). Unveiling the Differences of Secretome of Human Bone Marrow Mesenchymal Stem Cells, adipose tissue-derived stem cells, and human umbilical cord perivascular cells: a proteomic analysis. Stem Cells Dev.

[48] Mennan C, Wright K, Bhattacharjee A, Balain B, Richardson J, Roberts S (2013). Isolation and characterisation of mesenchymal stem cells from different regions of the human umbilical cord. Biomed Res Int.

[49] Shang Y, Guan H, Zhou F. Biological characteristics of umbilical cord mesenchymal stem cells and its therapeutic potential for Hematological disorders. Front Cell Dev Biology 2021, 9. 10.3389/fcell.2021.570179 PMC8126649 34012958

[50] Zhao D, Yu Z, Li Y, Wang Y, Li Q, Han D (2020). GelMA combined with sustained release of HUVECs derived exosomes for promoting cutaneous wound healing and facilitating skin regeneration. J Mol Hist.

[51] Zhang B, Wu X, Zhang X, Sun Y, Yan Y, Shi H, Zhu Y, Wu L, Pan Z, Zhu W (2015). Human umbilical cord mesenchymal stem cell exosomes Enhance Angiogenesis through the Wnt4/β-Catenin pathway. Stem Cells Translational Medicine.

[52] Henriques-Antunes H, Cardoso RMS, Zonari A, Correia J, Leal EC, Jiménez-Balsa A, Lino MM, Barradas A, Kostic I, Gomes C (2019). The kinetics of small extracellular vesicle delivery impacts skin tissue regeneration. ACS Nano.

[53] Tan SHS, Wong JRY, Sim SJY, Tjio CKE, Wong KL, Chew JRJ, Hui JHP, Toh WS (2020). Mesenchymal stem cell exosomes in bone regenerative strategies—a systematic review of preclinical studies. Mater Today Bio.

[54] Yang Q, Nanayakkara GK, Drummer C, Sun Y, Johnson C, Cueto R, Fu H, Shao Y, Wang L, Yang WY (2017). Low-intensity Ultrasound-Induced anti-inflammatory effects are mediated by several new mechanisms including gene induction, Immunosuppressor Cell Promotion, and enhancement of Exosome Biogenesis and Docking. Front Physiol.

[55] Wang Y, Cao Z, Wei Q, Ma K, Hu W, Huang Q, Su J, Li H, Zhang C, Fu X (2022). VH298-loaded extracellular vesicles released from gelatin methacryloyl hydrogel facilitate diabetic wound healing by HIF-1α-mediated enhancement of angiogenesis. Acta Biomater.

[56] Jones BA, Pei M (2012). Synovium-derived stem cells: a tissue-specific stem cell for Cartilage Engineering and Regeneration. Tissue Eng Part B: Reviews.

[57] Tao S-C, Guo S-C, Li M, Ke Q-F, Guo Y-P, Zhang C-Q (2017). Chitosan Wound dressings incorporating exosomes derived from MicroRNA-126-Overexpressing synovium mesenchymal stem cells provide sustained release of exosomes and heal full-thickness skin defects in a Diabetic Rat Model. Stem Cells Translational Medicine.

[58] Kwak G, Cheng J, Kim H, Song S, Lee SJ, Yang Y, Jeong JH, Lee JE, Messersmith PB, Kim SH (2022). Sustained exosome-guided macrophage polarization using hydrolytically degradable PEG hydrogels for Cutaneous Wound Healing: identification of Key proteins and MiRNAs, and sustained release formulation. Small.

[59] Xu N, Wang L, Guan J, Tang C, He N, Zhang W, Fu S (2018). Wound healing effects of a Curcuma zedoaria polysaccharide with platelet-rich plasma exosomes assembled on chitosan/silk hydrogel sponge in a diabetic rat model. Int J Biol Macromol.

[60] Ramírez OJ, Alvarez S, Contreras-Kallens P, Barrera NP, Aguayo S, Schuh CMAP (2020). Type I collagen hydrogels as a delivery matrix for royal jelly derived extracellular vesicles. Drug Delivery.

[61] Hu N, Cai Z, Jiang X, Wang C, Tang T, Xu T, Chen H, Li X, Du X, Cui W (2023). Hypoxia-pretreated ADSC-derived exosome-embedded hydrogels promote angiogenesis and accelerate diabetic wound healing. Acta Biomater.

[62] Li M, Ke Q-F, Tao S-C, Guo S-C, Rui B-Y, Guo Y-P (2016). Fabrication of hydroxyapatite/chitosan composite hydrogels loaded with exosomes derived from mir-126-3p overexpressed synovial mesenchymal stem cells for diabetic chronic wound healing. J Mater Chem B.

[63] Portela R, Leal CR, Almeida PL, Sobral RG (2019). Bacterial cellulose: a versatile biopolymer for wound dressing applications. Microb Biotechnol.

[64] Zhong SP, Zhang YZ, Lim CT (2010). Tissue scaffolds for skin wound healing and dermal reconstruction. WIREs Nanomed Nanobiotechnol.

[65] Dai N-T, Williamson MR, Khammo N, Adams EF, Coombes AGA (2004). Composite cell support membranes based on collagen and polycaprolactone for tissue engineering of skin. Biomaterials.

[66] Shafei S, Khanmohammadi M, Heidari R, Ghanbari H, Nooshabadi VT, Farzamfar S, Akbariqomi M, Sanikhani NS, Absalan M, Tavoosidana G (2020). Exosome loaded alginate hydrogel promotes tissue regeneration in full-thickness skin wounds: an in vivo study. J Biomedical Mater Res Part A.

[67] Zhu W, Dong Y, Xu P, Pan Q, Jia K, Jin P, Zhou M, Xu Y, Guo R, Cheng B (2022). A composite hydrogel containing resveratrol-laden nanoparticles and platelet-derived extracellular vesicles promotes wound healing in diabetic mice. Acta Biomater.

[68] Ferroni L, Gardin C, D’Amora U, Calzà L, Ronca A, Tremoli E, Ambrosio L, Zavan B (2022). Exosomes of mesenchymal stem cells delivered from methacrylated hyaluronic acid patch improve the regenerative properties of endothelial and dermal cells. Biomaterials Adv.

[69] Liu H, Wu B, Shi X, Cao Y, Zhao X, Liang D, Qin Q, Liang X, Lu W, Wang D, Liu J. Aerobic exercise-induced circulating extracellular vesicle combined decellularized dermal matrix hydrogel facilitates diabetic wound healing by promoting angiogenesis. Front Bioeng Biotechnol 2022, 10. 10.3389/fbioe.2022.903779 PMC9445842 36082169

[70] Zhang X, Gan J, Fan L, Luo Z, Zhao Y (2023). Bioinspired Adaptable Indwelling Microneedles for Treatment of Diabetic Ulcers. Adv Mater.

[71] Jiang T, Liu S, Wu Z, Li Q, Ren S, Chen J, Xu X, Wang C, Lu C, Yang X, Chen Z. ADSC-exo@MMP-PEG smart hydrogel promotes diabetic wound healing by optimizing cellular functions and relieving oxidative stress. Mater Today Bio 2022, 16. 10.1016/j.mtbio.2022.100365 PMC9364034 35967739

[72] Bari E, Scocozza F, Perteghella S, Sorlini M, Auricchio F, Torre ML, Conti M (2021). 3D Bioprinted scaffolds containing mesenchymal Stem/Stromal lyosecretome: Next Generation Controlled Release device for bone regenerative medicine. Pharmaceutics.

[73] Yang J, Chen Z, Pan D, Li H, Shen J (2020). Umbilical cord-derived mesenchymal stem cell-derived Exosomes Combined Pluronic F127 Hydrogel Promote Chronic Diabetic Wound Healing and Complete skin regeneration. IJN.

[74] Antezana PE, Municoy S, Alvarez-Echazu MI, Santo-Orihuela PL, Catalano PN, Al-Tel TH, Kadumudi FB, Dolatshahi-Pirouz A, Orive G, Desimone MF. The 3D Bioprinted scaffolds for Wound Healing. Pharmaceutics 2022, 14. 10.3390/pharmaceutics14020464 PMC8875365 35214197

[75] Gonzalez-Fernandez T, Tenorio AJ, Campbell KT, Silva EA, Leach JK (2021). Alginate-based bioinks for 3D bioprinting and fabrication of anatomically accurate bone grafts. Tissue Eng Part A.

[76] Varaprasad K, Jayaramudu T, Kanikireddy V, Toro C, Sadiku ER (2020). Alginate-based composite materials for wound dressing application:a mini review. Carbohydr Polym.

[77] Mndlovu H, du Toit LC, Kumar P, Marimuthu T, Kondiah PPD, Choonara YE, Pillay V (2019). Development of a fluid-absorptive alginate-chitosan bioplatform for potential application as a wound dressing. Carbohydr Polym.

[78] Zhou J, Du X, Chen X, Xu B (2018). Adaptive multifunctional supramolecular assemblies of Glycopeptides rapidly enable morphogenesis. Biochemistry.

[79] Bai XP, Zheng HX, Fang R, Wang TR, Hou XL, Li Y, Chen XB, Tian WM (2011). Fabrication of engineered heart tissue grafts from alginate/collagen barium composite microbeads. Biomed Mater.

[80] Cui B, Zhang C, Gan B, Liu W, Liang J, Fan Z, Wen Y, Yang Y, Peng X, Zhou Y (2020). Collagen-tussah silk fibroin hybrid scaffolds loaded with bone mesenchymal stem cells promote skin wound repair in rats. Mater Sci Eng C Mater Biol Appl.

[81] Chen XB, Fazel Anvari-Yazdi A, Duan X, Zimmerling A, Gharraei R, Sharma NK, Sweilem S, Ning L (2023). Biomaterials / bioinks and extrusion bioprinting. Bioact Mater.

[82] Masri S, Zawani M, Zulkiflee I, Salleh A, Fadilah NIM, Maarof M, Wen APY, Duman F, Tabata Y, Aziz IA et al. Cellular Interaction of Human skin cells towards natural bioink via 3D-Bioprinting technologies for Chronic Wound: a Comprehensive Review. Int J Mol Sci 2022, 23. 10.3390/ijms23010476 PMC8745539 35008902

[83] Lazaridou M, Bikiaris DN, Lamprou DA. 3D Bioprinted Chitosan-based hydrogel scaffolds in tissue Engineering and Localised Drug Delivery. Pharmaceutics; 2022. p. 14. 10.3390/pharmaceutics14091978 PMC9500618 36145727

[84] Qu J, Zhao X, Liang Y, Zhang T, Ma PX, Guo B (2018). Antibacterial adhesive injectable hydrogels with rapid self-healing, extensibility and compressibility as wound dressing for joints skin wound healing. Biomaterials.

[85] Qiao Z, Lv X, He S, Bai S, Liu X, Hou L, He J, Tong D, Ruan R, Zhang J (2021). A mussel-inspired supramolecular hydrogel with robust tissue anchor for rapid hemostasis of arterial and visceral bleedings. Bioact Mater.

[86] Endo Y, Yoshida H, Ota Y, Akazawa Y, Sayo T, Hanai U, Imagawa K, Sasaki M, Takahashi Y (2021). Accelerated human epidermal turnover driven by increased hyaluronan production. J Dermatol Sci.

[87] Feng P, Luo Y, Ke C, Qiu H, Wang W, Zhu Y, Hou R, Xu L, Wu S (2021). Chitosan-based functional materials for skin wound repair: mechanisms and applications. Front Bioeng Biotechnol.

[88] Maiz-Fernandez S, Barroso N, Perez-Alvarez L, Silvan U, Vilas-Vilela JL, Lanceros-Mendez S (2021). 3D printable self-healing hyaluronic acid/chitosan polycomplex hydrogels with drug release capability. Int J Biol Macromol.

[89] Coskun S, Akbulut SO, Sarikaya B, Cakmak S, Gumusderelioglu M (2022). Formulation of chitosan and chitosan-nanoHAp bioinks and investigation of printability with optimized bioprinting parameters. Int J Biol Macromol.

[90] Ahmed J, Gultekinoglu M, Edirisinghe M (2020). Bacterial cellulose micro-nano fibres for wound healing applications. Biotechnol Adv.

[91] Hickey RJ, Pelling AE (2019). Cellulose biomaterials for tissue Engineering. Front Bioeng Biotechnol.

[92] Alven S, Aderibigbe BA. Chitosan and Cellulose-based hydrogels for Wound Management. Int J Mol Sci 2020, 21. 10.3390/ijms21249656 PMC7767230 33352826

[93] Wang X, Wang Q, Xu C. Nanocellulose-based inks for 3D bioprinting: key aspects in Research Development and Challenging perspectives in Applications-A Mini Review. Bioeng (Basel) 2020, 7. 10.3390/bioengineering7020040 PMC7355978 32365578

[94] Chouhan D, Mandal BB (2020). Silk biomaterials in wound healing and skin regeneration therapeutics: from bench to bedside. Acta Biomater.

[95] Farokhi M, Mottaghitalab F, Fatahi Y, Khademhosseini A, Kaplan DL (2018). Overview of Silk Fibroin Use in Wound Dressings. Trends Biotechnol.

[96] Gholipourmalekabadi M, Sapru S, Samadikuchaksaraei A, Reis RL, Kaplan DL, Kundu SC (2020). Silk fibroin for skin injury repair: where do things stand?. Adv Drug Deliv Rev.

[97] Kundu B, Rajkhowa R, Kundu SC, Wang X (2013). Silk fibroin biomaterials for tissue regenerations. Adv Drug Deliv Rev.

[98] Kim E, Seok JM, Bae SB, Park SA, Park WH (2021). Silk Fibroin enhances Cytocompatibilty and Dimensional Stability of Alginate Hydrogels for light-based three-Dimensional Bioprinting. Biomacromolecules.

[99] Netti F, Aviv M, Dan Y, Rudnick-Glick S, Halperin-Sternfeld M, Adler-Abramovich L (2022). Stabilizing gelatin-based bioinks under physiological conditions by incorporation of ethylene-glycol-conjugated Fmoc-FF peptides. Nanoscale.

[100] Amondarain M, Gallego I, Puras G, Saenz-Del-Burgo L, Luzzani C, Pedraz JL (2023). The role of microfluidics and 3D-bioprinting in the future of exosome therapy. Trends Biotechnol.

[101] Gungor-Ozkerim PS, Inci I, Zhang YS, Khademhosseini A, Dokmeci MR (2018). Bioinks for 3D bioprinting: an overview. Biomater Sci.

[102] Nuutila K, Samandari M, Endo Y, Zhang Y, Quint J, Schmidt TA, Tamayol A, Sinha I (2022). In vivo printing of growth factor-eluting adhesive scaffolds improves wound healing. Bioact Mater.

[103] Xu Y, Xu C, He L, Zhou J, Chen T, Ouyang L, Guo X, Qu Y, Luo Z, Duan D (2022). Stratified-structural hydrogel incorporated with magnesium-ion-modified black phosphorus nanosheets for promoting neuro-vascularized bone regeneration. Bioact Mater.

[104] Abatangelo G, Vindigni V, Avruscio G, Pandis L, Brun P. Hyaluronic Acid: redefining its role. Cells 2020, 9. 10.3390/cells9071743 PMC7409253 32708202

[105] Rajaram A, Schreyer DJ, Chen DX (2015). Use of the polycation polyethyleneimine to improve the physical properties of alginate-hyaluronic acid hydrogel during fabrication of tissue repair scaffolds. J Biomater Sci Polym Ed.

[106] Little CJ, Kulyk WM, Chen X (2014). The Effect of Chondroitin Sulphate and Hyaluronic Acid on chondrocytes cultured within a fibrin-alginate hydrogel. J Funct Biomater.

[107] Li C, Zheng Z, Jia J, Zhang W, Qin L, Zhang W, Lai Y (2022). Preparation and characterization of photocurable composite extracellular matrix-methacrylated hyaluronic acid bioink. J Mater Chem B.

[108] Jorgensen AM, Chou Z, Gillispie G, Lee SJ, Yoo JJ, Soker S, Atala A. Decellularized skin extracellular matrix (dsECM) improves the Physical and Biological Properties of Fibrinogen Hydrogel for skin bioprinting applications. Nanomaterials (Basel) 2020, 10. 10.3390/nano10081484 PMC7466410 32751101

[109] Debels H, Hamdi M, Abberton K, Morrison W (2015). Dermal matrices and bioengineered skin substitutes: a critical review of current options. Plast Reconstr Surg Glob Open.

[110] Jang KS, Park SJ, Choi JJ, Kim HN, Shim KM, Kim MJ, Jang IH, Jin SW, Kang SS, Kim SE, Moon SH. Therapeutic efficacy of Artificial skin produced by 3D bioprinting. Mater (Basel) 2021, 14. 10.3390/ma14185177 PMC8467964 34576409

[111] Dzobo K, Motaung K, Adesida A. Recent trends in Decellularized Extracellular Matrix Bioinks for 3D Printing: an updated review. Int J Mol Sci 2019, 20. 10.3390/ijms20184628 PMC6788195 31540457

[112] Han Z, Dong L, Li A, Li Z, Fu L, Zhang Z, Li X, Li X (2022). Efficient angiogenesis-based wound healing through hydrogel dressing with extracellular vesicles release. Mater Today Bio.

[113] Nooshabadi VT, Khanmohamadi M, Valipour E, Mahdipour S, Salati A, Malekshahi ZV, Shafei S, Amini E, Farzamfar S, Ai J (2020). Impact of exosome-loaded chitosan hydrogel in wound repair and layered dermal reconstitution in mice animal model. J Biomedical Mater Res Part A.

[114] Wang M, Wang C, Chen M, Xi Y, Cheng W, Mao C, Xu T, Zhang X, Lin C, Gao W (2019). Efficient angiogenesis-based Diabetic Wound Healing/Skin Reconstruction through Bioactive Antibacterial Adhesive Ultraviolet shielding nanodressing with Exosome Release. ACS Nano.

[115] Wang C, Liang C, Wang R, Yao X, Guo P, Yuan W, Liu Y, Song Y, Li Z, Xie X (2019). The fabrication of a highly efficient self-healing hydrogel from natural biopolymers loaded with exosomes for the synergistic promotion of severe wound healing. Biomater Sci.

[116] Zhou Y, Zhang X-L, Lu S-T, Zhang N-Y, Zhang H-J, Zhang J, Zhang J (2022). Human adipose-derived mesenchymal stem cells-derived exosomes encapsulated in pluronic F127 hydrogel promote wound healing and regeneration. Stem Cell Res Ther.

[117] Bar A, Kryukov O, Etzion S, Cohen S (2023). Engineered extracellular vesicle-mediated delivery of miR-199a-3p increases the viability of 3D-printed cardiac patches. Int J Bioprinting.

[118] Bari E, Gravina GM, Scocozza F, Perteghella S, Frongia B, Tengattini S, Segale L, Torre ML, Conti M. Silk Fibroin Bioink for 3D Printing in tissue regeneration: controlled release of MSC extracellular vesicles. Pharmaceutics 2023, 15. 10.3390/pharmaceutics15020383 PMC9959026 36839705

[119] Thomas V, Yallapu MM, Sreedhar B, Bajpai SK (2009). Breathing-in/breathing-out approach to preparing nanosilver-loaded hydrogels: highly efficient antibacterial nanocomposites. J Appl Polym Sci.

[120] Tang L, Zhao C, Liu Y, Zhou J, Dong Y, Huang J, Yang T, Xiao H, Liu D, Wang S, Cai H (2023). GelMA Hydrogel loaded with extracellular vesicles derived from umbilical cord mesenchymal stem cells for promoting Cutaneous Diabetic Wound Healing. ACS Omega.

[121] Chen Y-C, Lin R-Z, Qi H, Yang Y, Bae H, Melero-Martin JM, Khademhosseini A (2012). Functional Human Vascular Network Generated in Photocrosslinkable Gelatin Methacrylate Hydrogels. Adv Funct Mater.

[122] Xu L, Liu Y, Tang L, Xiao H, Yang Z, Wang S (2022). Preparation of recombinant human collagen III protein hydrogels with sustained release of Extracellular vesicles for skin Wound Healing. Int J Mol Sci.

[123] Shitrit Y, Davidovich-Pinhas M, Bianco-Peled H (2019). Shear thinning pectin hydrogels physically cross-linked with chitosan nanogels. Carbohydr Polym.

[124] Palmara G, Frascella F, Roppolo I, Chiappone A, Chiado A (2021). Functional 3D printing: approaches and bioapplications. Biosens Bioelectron.

[125] Li Q, Yu H, Zhao F, Cao C, Wu T, Fan Y, Ao Y, Hu X (2023). 3D Printing of Microenvironment-Specific Bioinspired and Exosome-Reinforced Hydrogel scaffolds for efficient cartilage and subchondral bone regeneration. Adv Sci (Weinh).

[126] Huang J, Yang R, Jiao J, Li Z, Wang P, Liu Y, Li S, Chen C, Li Z, Qu G (2023). A click chemistry-mediated all-peptide cell printing hydrogel platform for diabetic wound healing. Nat Commun.

[127] Lee J, Dutta SD, Acharya R, Park H, Kim H, Randhawa A, Patil TV, Ganguly K, Luthfikasari R, Lim KT. Stimuli-responsive 3D printable conductive hydrogel: a step toward regulating macrophage polarization and Wound Healing. Adv Healthc Mater 2023:e2302394. 10.1002/adhm.202302394 37950552

[128] Kim BS, Kwon YW, Kong J-S, Park GT, Gao G, Han W, Kim M-B, Lee H, Kim JH, Cho D-W (2018). 3D cell printing of in vitro stabilized skin model and in vivo pre-vascularized skin patch using tissue-specific extracellular matrix bioink: a step towards advanced skin tissue engineering. Biomaterials.

[129] Zhong Y, Ma H, Lu Y, Cao L, Cheng YY, Tang X, Sun H, Song K (2023). Investigation on repairing diabetic foot ulcer based on 3D bio-printing Gel/dECM/Qcs composite scaffolds. Tissue Cell.

[130] Born LJ, McLoughlin ST, Dutta D, Mahadik B, Jia X, Fisher JP, Jay SM (2022). Sustained released of bioactive mesenchymal stromal cell-derived extracellular vesicles from 3D-printed gelatin methacrylate hydrogels. J Biomed Mater Res A.

[131] Su N, Hao Y, Wang F, Hou W, Chen H, Luo Y. Mesenchymal stromal exosome-functionalized scaffolds induce innate and adaptive immunomodulatory responses toward tissue repair. Sci Adv 2021, 7. 10.1126/sciadv.abf7207 PMC8115917 33980490

[132] Altabas V. Diabetes, Endothelial Dysfunction, and Vascular Repair: What Should a Diabetologist Keep His Eye on? *Int J Endocrinol* 2015, 2015:848272. 10.1155/2015/848272 PMC4452196 26089898

[133] Telgenhoff D, Shroot B (2005). Cellular senescence mechanisms in chronic wound healing. Cell Death Differ.

[134] Wall IB, Moseley R, Baird DM, Kipling D, Giles P, Laffafian I, Price PE, Thomas DW, Stephens P (2008). Fibroblast dysfunction is a key factor in the non-healing of chronic venous Leg Ulcers. J Invest Dermatol.

[135] Tsourdi E, Barthel A, Rietzsch H, Reichel A, Bornstein SR (2013). Current aspects in the pathophysiology and treatment of chronic wounds in diabetes mellitus. Biomed Res Int.

[136] Xue M, Jackson CJ (2015). Extracellular matrix reorganization during Wound Healing and its impact on abnormal scarring. Adv Wound Care.

[137] Wang Y, Song P, Wu L, Su Z, Gui X, Gao C, Zhao H, Wang Y, Li Z, Cen Y (2023). In situ photo-crosslinked adhesive hydrogel loaded with mesenchymal stem cell-derived extracellular vesicles promotes diabetic wound healing. J Mater Chem B.

[138] Coentro JQ, Pugliese E, Hanley G, Raghunath M, Zeugolis DI (2019). Current and upcoming therapies to modulate skin scarring and fibrosis. Adv Drug Deliv Rev.

[139] Shen Y, Xu G, Huang H, Wang K, Wang H, Lang M, Gao H, Zhao S (2021). Sequential release of small extracellular vesicles from Bilayered Thiolated Alginate/Polyethylene Glycol Diacrylate Hydrogels for Scarless Wound Healing. ACS Nano.

[140] Kim SY, Nair MG (2019). Macrophages in wound healing: activation and plasticity. Immunol Cell Biol.

[141] Barbay V, Houssari M, Mekki M, Banquet S, Edwards-Levy F, Henry JP, Dumesnil A, Adriouch S, Thuillez C, Richard V, Brakenhielm E (2015). Role of M2-like macrophage recruitment during angiogenic growth factor therapy. Angiogenesis.

[142] Shook B, Xiao E, Kumamoto Y, Iwasaki A, Horsley V (2016). CD301b + macrophages are essential for effective skin Wound Healing. J Invest Dermatol.

[143] Schilling JA (1976). Wound healing. Surg Clin North Am.

[144] Ji S, Zhu Z, Sun X, Fu X (2021). Functional hair follicle regeneration: an updated review. Sig Transduct Target Ther.

[145] Huang C, Du Y, Nabzdyk CS, Ogawa R, Koyama T, Orgill DP, Fu X (2016). Regeneration of hair and other skin appendages: a microenvironment-centric view. Wound Repair and Regeneration.

[146] Bao H, Pan Y, Ping Y, Sahoo NG, Wu T, Li L, Li J, Gan LH (2011). Chitosan-Functionalized Graphene Oxide as a Nanocarrier for Drug and Gene Delivery. Small.

[147] Huang S, Ge X, Yu J, Han Z, Yin Z, Li Y, Chen F, Wang H, Zhang J, Lei P (2018). Increased mir-124-3p in microglial exosomes following traumatic brain injury inhibits neuronal inflammation and contributes to neurite outgrowth via their transfer into neurons. FASEB J.

[148] Qian Z, Bai Y, Zhou J, Li L, Na J, Fan Y, Guo X, Liu H (2020). A moisturizing chitosan-silk fibroin dressing with silver nanoparticles-adsorbed exosomes for repairing infected wounds. J Mater Chem B.

[149] Pop-Busui R, Ang L, Holmes C, Gallagher K, Feldman EL (2016). Inflammation as a therapeutic target for Diabetic neuropathies. Curr Diab Rep.

[150] Buschmann D, Mussack V, Byrd JB (2021). Separation, characterization, and standardization of extracellular vesicles for drug delivery applications. Adv Drug Deliv Rev.

[151] Gandham S, Su X, Wood J, Nocera AL, Alli SC, Milane L, Zimmerman A, Amiji M, Ivanov AR (2020). Technologies and standardization in Research on Extracellular vesicles. Trends Biotechnol.

[152] Gorgens A, Corso G, Hagey DW, Jawad Wiklander R, Gustafsson MO, Felldin U, Lee Y, Bostancioglu RB, Sork H, Liang X (2022). Identification of storage conditions stabilizing extracellular vesicles preparations. J Extracell Vesicles.

[153] Negut I, Dorcioman G, Grumezescu V. Scaffolds for Wound Healing Applications. Polymers 2020, 12. 10.3390/polym12092010 PMC7563417 32899245

[154] Bakaic E, Smeets NMB, Hoare T (2015). Injectable hydrogels based on poly(ethylene glycol) and derivatives as functional biomaterials. RSC Adv.

[155] Yue K, Santiago GT-d, Alvarez MM, Tamayol A, Annabi N, Khademhosseini A (2015). Synthesis, properties, and biomedical applications of gelatin methacryloyl (GelMA) hydrogels. Biomaterials.

[156] Yu C, Schimelman J, Wang P, Miller KL, Ma X, You S, Guan J, Sun B, Zhu W, Chen S (2020). Photopolymerizable Biomaterials and Light-based 3D Printing Strategies for Biomedical Applications. Chem Rev.

[157] Murphy SV, Atala A (2014). 3D bioprinting of tissues and organs. Nat Biotechnol.

[158] Blaeser A, Campos DFD, Puster U, Richtering W, Stevens MM, Fischer H (2016). Controlling Shear stress in 3D bioprinting is a key factor to Balance Printing Resolution and Stem Cell Integrity. Adv Healthc Mater.

[159] Placone JK, Engler AJ (2018). Recent advances in extrusion-based 3D Printing for Biomedical Applications. Adv Healthc Mater.

[160] Jin Z, Zhang Z, Shao X, Gu GX (2023). Monitoring anomalies in 3D bioprinting with deep neural networks. ACS Biomater Sci Eng.

[161] Lee J, Oh SJ, An SH, Kim WD, Kim SH (2020). Machine learning-based design strategy for 3D printable bioink: elastic modulus and yield stress determine printability. Biofabrication.

